# All three quinone species play distinct roles in ensuring optimal growth under aerobic and fermentative conditions in *E*. *coli* K12

**DOI:** 10.1371/journal.pone.0194699

**Published:** 2018-04-03

**Authors:** Annika Nitzschke, Katja Bettenbrock

**Affiliations:** Max-Planck-Institute for Dynamics of Complex Technical Systems, Sandtorstraße, Magdeburg, Germany; University of Alberta, CANADA

## Abstract

The electron transport chain of *E*. *coli* contains three different quinone species, ubiquinone (UQ), menaquinone (MK) and demethylmenaquinone (DMK). The content and ratio of the different quinone species vary depending on the external conditions. To study the function of the different quinone species in more detail, strains with deletions preventing UQ synthesis, as well as MK and/or DMK synthesis were cultured under aerobic and anaerobic conditions. The strains were characterized with respect to growth and product synthesis. As quinones are also involved in the control of ArcB/A activity, we analyzed the phosphorylation state of the response regulator as well as the expression of selected genes.The data show reduced aerobic growth coupled to lactate production in the mutants defective in ubiquinone synthesis. This confirms the current assumption that ubiquinone is the main quinone under aerobic growth conditions. In the UQ mutant strains the amount of MK and DMK is significantly elevated. The strain synthesizing only DMK is less affected in growth than the strain synthesizing MK as well as DMK. An inhibitory effect of MK on aerobic growth due to increased oxidative stress is postulated.Under fermentative growth conditions the mutant synthesizing only UQ is severely impaired in growth. Obviously, UQ is not able to replace MK and DMK during anaerobic growth.

Mutations affecting quinone synthesis have an impact on ArcA phosphorylation only under anaerobic conditions. ArcA phosphorylation is reduced in strains synthesizing only MK or MK plus DMK.

## Introduction

*E*. *coli* is a facultative anaerobic bacterium. Therefore, it needs to be able to adapt its metabolism to different oxygen concentrations. When oxygen is available, it acts as the preferred electron acceptor. It is used to reoxidize NADH to NAD^+^ through the respiratory electron transport chain, which is accompanied by ATP production [1]. If no oxygen or alternative electron acceptor is available, the reducing equivalents are regenerated by the formation of lactate, ethanol, formate and succinate. Together with acetate these are the products of mixed acid fermentation [2, 3].

The adaptation between fermentation and respiration is controlled by two main transcription factors: the cytoplasmic transcription factor FNR (fumarate and nitrate reduction) and the two-component system ArcB/A (aerobic respiration control) [4–9]. FNR is active only in the absence of oxygen. In the presence of oxygen an iron-sulfur-cluster is oxidized and thereby FNR is inactivated [10–12]. In contrast to FNR, the ArcB/A system reacts only indirectly to changes in the oxygen supply [13]. It consists of the membrane linked sensor kinase ArcB and the cytoplasmic response regulator ArcA [14]. ArcB is an atypical sensor kinase that consists of an input domain with a functional leucine zipper and a PAS domain and of a catalytic domain, which contains a transmitter domain, a central receiver domain and C-terminal phospho-transfer domain. When the oxygen supply is increased, disulfide bonds form in the PAS domain and ArcB is inactivated [15]. Hence, under anaerobic and microaerobic conditions ArcB undergoes autophosphorylation [16]. Phosphorylated ArcB catalyzes the transphosphorylation of ArcA that controls genes of the Tricarboxylic Acid Cycle (TCA) and of mixed acid fermentation [14, 17]. Under oxidizing conditions, ArcB dephosphorylates ArcA-P through a reverse phosphorelay [18].

Currently it is assumed that the redox level of the quinone pool is responsible for the activation of the sensor kinase ArcB [13, 17, 19]. Quinones function as membrane bound electron carriers between dehydrogenases and oxidases in the aerobic and anaerobic respiratory chains. *E*. *coli* synthesizes three different kinds of quinones: ubiquinone (UQ; E'^0^ = +113 mV), menaquinone (MK; E'^0^ = -74 mV) and demethylmenaquinone (DMK, E'^0^ = +36 mV). The different quinone species differ in their redox potential and interact with different enzymes. Due to its high redox potential, UQ operates mainly as part of the aerobic respiratory chain, transferring electrons to the terminal oxidases [20–24]. MK and DMK are preferentially synthesized under anaerobic to microaerobic conditions [1, 25, 26]. Electrons are transferred to fumarate reductase by MK or DMK, while MK or UQ transfer electrons to nitrate reductase [1, 27, 28].

There are several, in part contradictory publications concerning ArcB activation. Alvarez and coworkers assumed that under aerobic conditions the electrons from the cysteine residues of ArcB are able to reduce UQ [16] resulting in disulfide bond formation and immediate silencing of ArcB kinase activity. During the transition from microaerobic to anaerobic conditions, the low redox potential of MK permits electron transfer from reduced MK to the cysteine residues of ArcB, resulting in disulfide bond breakage and activation of the ArcB kinase activity. In a contradicting study Bekker and coworkers postulated that ArcA is activated under anaerobic (0% aerobiosis) and subaerobic (80% aerobiosis) conditions by reduced MK and/or UQ and is much less active under fully aerobic (100% aerobiosis) and microaerobic (20% aerobiosis) conditions [19]. In addition to quinones, organic acids e.g. lactate can modify ArcA activity [29, 30].

While the function of the different quinone species has been well investigated for aerobic and anaerobic respiration [24], there are almost no studies about their function under fermentative conditions. It is possible to construct strains that lack one or two quinone species by mutating specific enzymes. In a previous study, Sharma and coworkers analyzed such quinone mutants with respect to aerobic and anaerobic growth properties. They also analyzed quinone amounts and ArcA phosphorylation [31]. We performed experiments under aerobic as well as fermentative conditions, extending those studies by analysis of by-products and expression of selected genes. Notably, in these experiments we used a defined mineral medium containing glucose as sole carbon source, while Sharma and coworkers [31] added 1% LB to all cultures. While some of our data are in good agreement with those reported by Sharma and coworkers others are not. It is tempting to speculate that the addition of LB is the reason for these deviations.

We systematically investigated metabolism, gene expression and ArcA phosphorylation utilizing quinone mutants under aerobic as well as fermentative conditions. In contrast to indirect determination of the ArcA activity by reporter genes, the ArcA phosphorylation state was determined directly by Phos-tag gel electrophoresis and Western blotting. The data confirm the important function of UQ for aerobic respiration. They also show that DMK is able to replace UQ to a certain extent in respiration. Notably, the data indicate that the presence of high concentrations of MK under aerobic conditions is unfavorable for growth. Under anaerobic conditions, growth and product excretion of the mutants containing DMK or DMK plus MK is similar to the wild type while growth of the strain lacking UQ is strongly inhibited. This can also be seen in the gene expression data derived from growth under aerobic and anaerobic conditions.

## Materials and methods

### Bacterial strains and culture conditions

All strains are derived from *E*. *coli* K12 MG1655. They have defects in the ubiquinone synthesis (AV33), demethylmenaquinone and menaquinone synthesis (AV34) or in ubiquinone and menaquinone synthesis (AV36) [Table 1].

**Table 1:** Strains used in this study.

*E.coli K12*	*Genotyp*	*Quinone type present*	*Reference*
**MG1655**	wild type	UQ, DMK, MK	
**AV33**	Δ*ubiCA*	DMK,MK	[31]
**AV34**	Δ*menA*	UQ	[31]
**AV36**	Δ*ubiE*	DMK	[31]

For cultivations under aerobic and anaerobic growth conditions a single colony from LB_0_ plates was inoculated in 5 ml LB_0_ (10 g/l tryptone, 10 g/l yeast extract, 5 g/l NaCl) and cultivated for 5 h at 37°C. Evans medium with sodium phosphate buffer (100 mM, pH 7) and approximately 20 mM glucose inoculated with 1% of the first pre-culture was used as second pre-culture [32, 33]. After incubation with shaking overnight (aerobic with 250 rpm and semi-aerobic with 75 rpm) the cells were centrifuged at 5000 x g for 20 min. For the main culture, washed cells of the second pre-culture were inoculated to OD_420nm_ of 0.2–0.3 corresponding to 1–1.5 x 10^8^ cells/ml in fresh Evans medium. Cells were grown at 37°C. For aerobic growth, cultures were grown in shake flasks with baffles, with volumes at least 10 times higher than the culture volume, under vigorous shaking (250 rpm). For anaerobic growth cultures were incubated in sealed glass vials.

### Measurements of biomass and extracellular metabolites

Biomass was determined by measuring the optical density at 420 nm. An OD_420nm_ of 1 corresponds to about 5 x 10^8^ cells/ml. Extracellular concentrations of formate, acetate, pyruvate, malate, fumarate, lactate and succinate were measured by HPLC (Agilent 1100 series LC/MSD). An Insertil ODS-3 (RP-18 100A, 250x4.6nm) column was equilibrated with 100% 0.1 M ammonium dihydrogen phosphate (pH 2.6) as mobile phase with a flow rate of 1 ml/min. The injection volume was 10 μL. Glucose and ethanol were determined enzymatically by using the D-glucose HK assay kit and the ethnol assay kit (Megazyme). Growth rates and yields were calculated from the data of at least three time points during exponential growth phase. Product yields were determined by plotting the respective product concentration against the corresponding glucose concentration at the different time points. The yield was determined by linear regression of the data points. At least three independent growth curves were analyzed. Time course data from all growth curves considered can be found in S1 Dataset (Suppl_data_aerobe.docx a_and Suppl_Data_anaerobe.docx).

### Quinone extraction analysis

The extraction and analysis of quinones was carried out essentially as described by Sharma [31]. 3 ml sample was added to 14 ml of a 1:1 mixture of methanol (quenching) and petroleum ether (for dissolving the quinones). The mixture was vortexed for 30 s and centrifuged for 5 min at 4500 x g. Afterwards, the petroleum ether phase was removed and transferred to a new glass tube. The petroleum ether was completely evaporated under a flow of nitrogen. The extract was stored at -20°C for no longer than 3 days.

Before measuring the samples by HPLC (Agilent 1100 Series LC/MSD; Detector 1946/1956 MSD) the extracts were re-suspended in 200 μL pure ethanol. The column (LiChrosorb 10-U (RP-18 100A, 250x4.6nm)) was equilibrated with pure methanol as mobile phase. Detection of ubiquinone was performed at 290 nm and of naphthoquinones at 248 nm. All quinone species can be quantified from the same run. The amounts of the quinones were calculated from the peak area by using UQ-10 and MK-4 as standards. The given concentrations refer to dry cell weight. This was calculated from the OD_420_ measurement with OD420 = 1 amounts to 0.21 g_DCW_/l.

All measured data from the experiments can be found in S1 Dataset (Suppl_data_Quinone_aerobe.docx and Suppl_data_Quinone_anaerobe.docx).

### ArcA phosphorylation measurement

The measurement of the relative ArcA phosphorylation was carried out by Phostag™-SDS gel electrophoresis [34] and Western blotting using rabbit ArcA polyclonal antiserum (kind gift of K. Hellingwerf, University of Amsterdam) as described by [29]. 5 ml of the culture was added to 1 ml formic acid (6 M) and 0.1 ml chloramphenicol (25 mg/ml). The sample was vortexed for 30 s and separated into 0.25 ml (aerobic) or 1 ml (anaerobic) aliquots. After that the samples were centrifuged at 4°C by 8000 rpm for 2 min and the supernatant was removed. The pellet was resuspended in 50 μL formic acid (1 M) and stored at -80°C.

A 10% acrylamide (w/v) resolving gel with 25 μM Phos-tag AAL (WAKO Chemicals) and 60 μM MnCl_2_ was prepared. For the upper part of the gel a 3% stacking gel was used. The samples were diluted to an end-concentration equal to 4.6 OD_420nm_ /ml in a mixture of 61.5% v/v formic acid (1 M), 33% (v/v) 3 x loading buffer and 5.5% (v/v) sodium hydroxide (10 M). 5 μL of each sample was loaded per well and 3 μL fluorescence Magic Mark western standard (Invitrogen) was loaded on the same gel. The gel was run at 12.5 mA. Afterwards, gels were washed in transfer buffer (methanol 20% (v/v), SDS 0.04% (w/v), Tris 0.3% (w/v), and glycine 1.5% (w/v)) for 15 min and after that in transfer buffer with EDTA (1 mM) again for 15 min. The gel was blotted for 20 min with 100 mA/gel onto a nitrocellulose membrane using a semi-dry blotting system.

### Gene expression analysis by RT-qPCR

About 1.5 x 10^9^ cells from exponential growth phase were quenched in twice the volume of RNAprotect Bacterial Reagent (Qiagen), vortexed for 5 s and incubated at room temperature for 5 min. Cells were pelleted by centrifugation, the supernatant was discarded and the pellet was stored at -80°C. RNA was prepared using the Master Pure RNA Purification Kit (Epicentre). RNA concentration and purity were determined using the NanoDrop spectrophotometer (Thermo Scientific). The mRNA was transcribed into cDNA by using the RevertAid H Minus First Strand cDNA synthesis Kit (Thermo Fisher Scientific). Quantitative PCR of different cDNA samples was performed using the MesaGreen qPCR Master Mix Plus (Eurogenetec) with SYBR Green as detection agent using a Rotor-Gene 6000 (Corbett Life Science). Amplification conditions were: 95°C for 10 min followed by 40 cycles of 95°C for 15 s and 60°C for 1 min. A negative control without template was conducted for each primer pair in each PCR run and a control for DNA contamination was performed for each RNA sample used. Primer sequences are indicated in Table 2. Quantification was performed by relative quantification to housekeeping genes (*rpoD*, *ihfB* and *recA*) applying the ΔΔCt method [35, 36] with efficiency correction.

**Table 2:** Primer sequences.

***Gene***	**Primer sequence (5'.....3')**
*adhE*	CACTCAATGGCGCACAAACTG
	GCCTGCGGACGGTCATACTGG
*frdA*	GCGCCGCGGGACTCTTTAC
	TGCGCATCACCGACACTTCC
*ldhA*	TGCGCTGTGCCGGTTTCAATAA
	CGGCGGTTCAGCGTCATCATC
*pflB*	GAGGCCCATACCACCGATAGAT
	AGAAGCGCAGGAAATGGTTGAC
*appC*	TGACCGGGGCCATGTTTATTAT
	GGTTGTACTTGCGCGACTTCAT
*cydA*	TGCGGCCTGTATACCCTGTTCC
	CGTGCCGGCTGAGTAGTCGTG
*mdh*	CCATTCTGCCGCTGCTGTCAC
	CCACCGGCCTTCGCTTCAA
*sdhD*	GATCGGTTTCTTCGCCTCTG
	CGGTCAACACCTGCCACAT
*cyoA*	CCGCTGGCACACGACGAGA
	AAGCGATTTCATTCACGGTAGCA
*ndh*	GTCGATCGTAACCACAGCCA
	GCATGGGCCAGATAGCTCAA
*nuoN*	TGTCGCGTTGGGTAAAAACC
	GAGAGAGTTTGAAGCCGAGGC
*poxB*	ATCATGCGCCACAACCAGTCGT
	ACCGCGCAGGGCATGAACAATA
*carA*	CCTGCGGATGCTGGTGGATAGA
	TGGATGGCGGTAATGGCGTAAT
*pyrB*	GCGCCTGGCCACCGAGTTTTC
	CGGTGCGGCCATATTTCAGGT
*pyrE*	CGGGCGCGGCGAGATTTC
	GCCTTAACCGCCGCCAGATGT
*ihfB*	GCCAAGACGGTTGAAGATGC
	GAGAAACTGCCGAAACCGC
*recA*	CGCTTGGGGCAGGTGGTCT
	TGCAGCGTCAGCGTGGTTTT
*rpoD*	TCTGCGTATGCGTTTCGGTATC
	ACGGCTCGGGTGACGCAGTT

### Determination of oxidative stress

To determine if mutant strains suffer from oxidative stress, flow cytometry with CellROX Green Reagent (Thermo Fisher Scientific) was applied, according to the protocol provided by the manufacturer and similar to the protocol of McBee [37]. After incubation of the second pre-culture without shaking overnight, the cells were centrifuged at 5000 x g for 20 min. For the main culture, washed cells of the second pre-culture were inoculated to OD_420_ of 0.1–0.2 in fresh Evans medium with 20 mM glucose. The cells were grown at 37°C in shake flasks with baffles with volumes at least 10 times higher than the culture volume under vigorous shaking (250 rpm). After approximately 4 h in the exponential phase 2 μl CellROX Green reagent (2.5 mM) was added to a 1 ml culture sample. Samples were incubated for 30 min at 37°C in a thermocycler with vigorous shaking at 700 rpm. Afterwards the mixture was centrifuged and the pellet was resuspended in a 1:1 mixture of FACS buffer (10 mM Tris, 10 mM MgCl_2_, pH 8.4 and 4% paraformaldehyde). Afterwards cells were stained with 1 μg/ml DAPI for 2–5 min at 37°C. To artificially induce oxidative stress, 100 μM menadione (Sigma) was added to the growing cultures 1 h before harvesting.

CellROX Green fluorescence was analyzed by flow cytometry in a CyFlow Space (Partec). Cells were gated by scatter parameters coupled to positive DAPI stain (FL4, 455/50). To compare different strains, mean CellRox Green fluorescence (FL1, 527/30) of the gated cells was determined using the analysis software of CyFlow Space. The measurement values of the different repeats can be found under S1 Dataset (Suppl_data_CellROX.docx).

## Results

### Growth and product formation

Different quinone species have been assigned different functions in metabolism, being important in respiration with O_2_ or alternative electron acceptors, respectively [1, 24]. So far, their function under fermentative conditions, i.e. during anaerobic growth without external electron acceptors, has not been investigated in detail. Also, different quinone species have been assigned different signaling properties in the activation and inactivation of the sensor kinase ArcB [16, 19, 29, 31, 38]. We used a set of strains [Table 1] that are defective in the synthesis of one or two of the three quinone species present in *E*. *coli*. These strains, as well as the parent strain MG1655, were grown in aerobic and anaerobic batch experiments in defined medium with glucose as sole carbon source. We analyzed growth, the production of by-products, the quinone content, ArcA phosphorylation as well as the expression of selected genes to get a comprehensive picture of the influence of the quinone content on strain physiology, thereby extending previous studies [31].

Firstly, the strains were grown under aerobic batch conditions. The two strains lacking UQ, AV33 and AV36, show significantly slower growth than the wild type strain and AV34, which produces UQ as sole quinone species [Table 3]. This shows the important function of UQ as electron carrier in the respiratory chain with oxygen as electron acceptor. The different growth rates of the mutants were accompanied by different product formation patterns. Aerobically, MG1655 and AV34 excreted only acetate, which is a known product in aerobic batch cultivations with glucose. Slow aerobic growth of AV33 and AV36 was accompanied by the production of significant amounts of lactate and low amounts of pyruvate [Table 3]. This is in agreement with previous analyses of UQ^-^ mutants [22, 23, 39]. Obviously, MK and DMK are not able to fully replace UQ in aerobic respiration, leading to a (partially) fermentative metabolism despite the presence of oxygen. To oxidize NADH in these strains, pyruvate is reduced to lactate by lactate dehydrogenase. In fact, AV33 excreted about 94% of the carbon taken up as organic acids and AV36 about 76%, compared to about 10% in MG1655. Notably, strain AV33 that produces MK as well as DMK grew even slower and produced more lactate than the DMK only mutant AV36 [Table 3].

**Table 3:** Aerobic growth rates and by-product yields of quinone mutants compared to MG1655.

AEROBIC	MG1655 _(UQ,DMK,MK)_	AV34 _(UQ)_	AV33 _(DMK,MK)_	AV36 _(DMK)_
Growth rate [h^-1^]	0.74 ± 0.07	0.75 ± 0.02	0.21 ± 0.02	0.38 ± 0.05
Yield [mol/mol_Glc_]				
Acetate	0.26 ± 0.08	0.29 ± 0.09	0.64 ± 0.12	0.61 ± 0.15
Lactate	N.D.	N.D.	1.45 ± 0.23	0.93 ± 0.25
Pyruvate	N.D.	N.D.	0.02 ± 0.0004	0.03 ± 0.01

The table indicates specific growth rates during the exponential phase of growth, given in doublings per hour. Product yields are indicated in mol product produced per mol of glucose consumed. N.D. in yields means that the respective product could not be detected throughout the time course of the measurements. Data are average values of at least three independent biological replicates.

**Fig 1:**
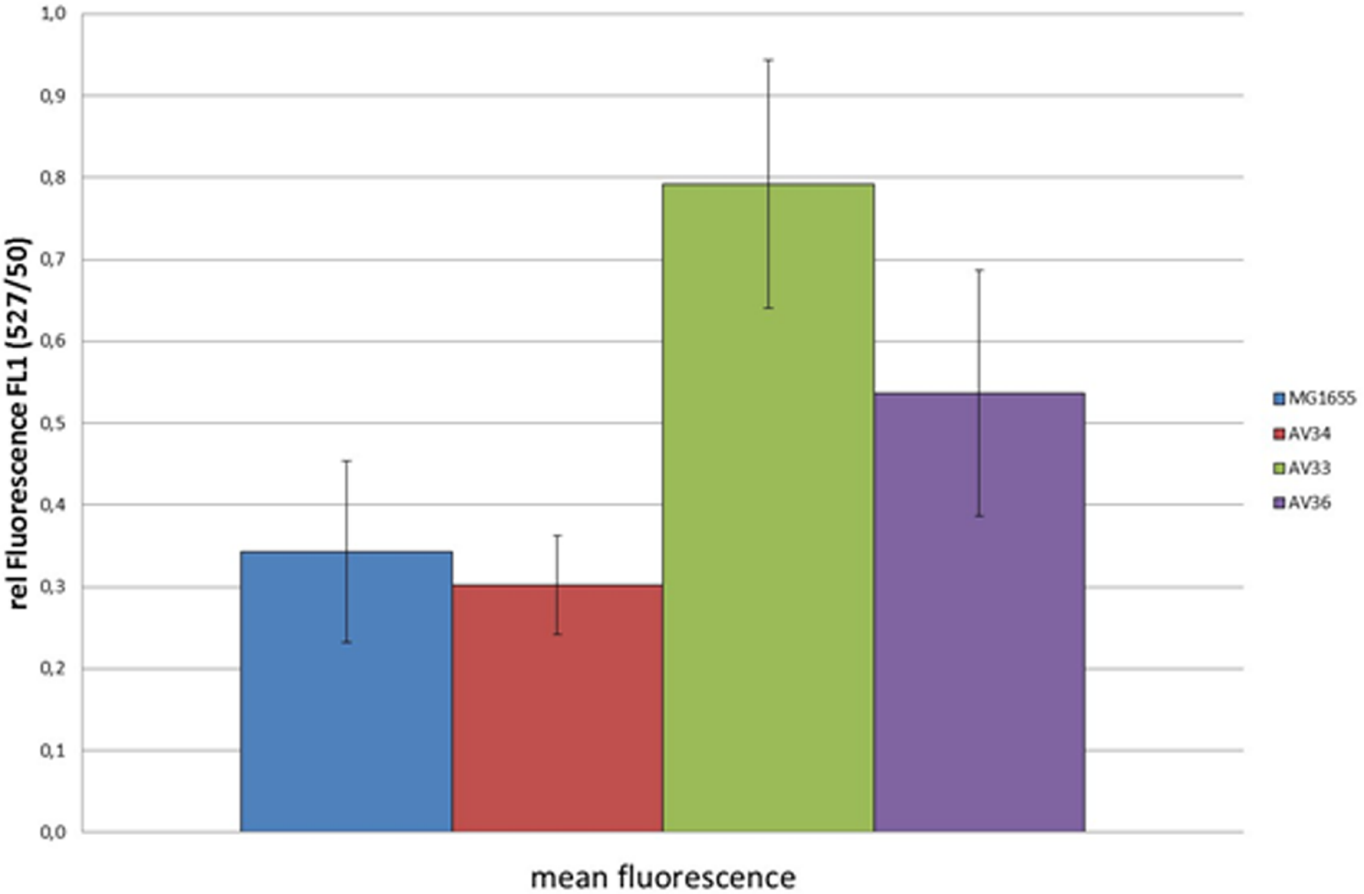
Reactive oxygen species in quinone mutants. Shown are mean fluorescence values of MG1655 and the three mutant strains after aerobic incubation with CellROX Green. Samples of aerobically growing cultures of the strains were taken from exponential growth phase and incubated with CellROX Green. The values represent average values from at least three independent growth curves.

Ubiquinone is known to protect *E*. *coli* from oxidative stress [40]. To analyze if the ubiquinone mutants suffer from increased oxidative stress, we analyzed oxidative stress in the different strains growing under aerobic conditions by flow cytometry, using CellROX Green as staining reagent. As can be seen from Fig 1, both AV33 and AV36, show increased CellROX Green fluorescence, indicating enhanced accumulation of superoxide anions, while AV34 and MG1655 showed only low fluorescence. Oxidative stress is especially pronounced in AV33, thereby reflecting the low growth rate of this strain. For comparison the strains were incubated with menadione, a superoxide anion generating analogue of MK [21]. Though CellROX Green fluorescence was higher in the menadione induced samples than in AV33 and AV36 (Fig A in S1 File), the fluorescence detected in those strains growing aerobically was clearly enhanced and hence hints to oxidative stress being elicited in these strains during aerobic growth.

Next, the strains were grown under anaerobic conditions in closed vials to analyze the impact of the different quinone species on fermentation. The UQ^-^ strains AV33 and AV36 show no significant differences in growth rate or product yields compared to MG1655. In contrast, AV34 containing UQ only, showed an extremely slow growth rate [Table 4]. The UQ only mutant showed similar acetate and ethanol production as the wild type but a lower amount of extracellular formate. Anaerobically, pyruvate is converted to acetyl-CoA and formate by the pyruvate-formate lyase (PFL) [3, 24, 41]. Acetyl-CoA reacts to either ethanol or acetate [2, 3]. A constant production of the acetyl-CoA derived products, ethanol and acetate, but a lower formate concentration hence could reflect enhanced cleavage of formate by formate hydrogen lyase (FHL) resulting in H_2_ and CO_2_ production [42]. Gaseous products have not been systematically analyzed in this study; however H_2_ and CO_2_ production by MG1655 and AV34 under fermentative conditions was followed in a bioreactor experiment with off-gas analysis to check for differences of both strains (Fig B in S1 File). Rates of H_2_ and CO_2_ production were not constant throughout the exponential growth phase, making it impossible to calculate precise rates. Nevertheless, from the plots it becomes obvious that, while both gases were produced in late exponential and stationary phase by MG1655, they were produced already in the exponential phase by AV34. The shift in the ratio between formate and ethanol /acetate could also be due to PDH being active in AV34. We hence determined PDH activity in cell extracts of MG1655 and AV34 growing anaerobically. Activities in both strains were extremely low (0.082 ± 0.008 U/(mg protein) for MG1655 and 0.057 ± 0.013 U/(mg protein) for AV34 but no obvious differences between both strains could be observed. AV34 also showed increased lactate formation. Notably, the determination of the lactate yield is complicated by variations in lactate production during exponential growth (Fig D in S1 File). Strikingly, AV34 did not produce the typical fermentation product succinate but produced a small amount of fumarate instead (Table 4). This confirms that the reaction of fumarate reductase is coupled to MK oxidation [1, 43].

**Table 4:** Anaerobic growth rates and by-product yields of all quinone mutants compared to MG1655.

ANAEROBIC	MG1655 _(UQ,DMK,MK)_	AV34 _(UQ)_	AV33 _(DMK,MK)_	AV36 _(DMK)_
Growth [h^-1^]	0.38 ± 0.07	0.05 ± 0.01	0.35 ± 0.02	0.37 ± 0.03
Yield [mol/mol_Glc_]				
Formate	1.21 ± 0.07	0.86 ± 0.20	1.32 ± 0.10	1.16 ± 0.14
Pyruvate	0.014 ± 0.002	0.03 ± 0.02	0.016 ± 0.004	0.012 ± 0.005
Lactate	0.39 ± 0.03	0.93 ± 0.03	0.32 ± 0.04	0.45 ± 0.11
DHO	0.0005±0.0001	0.029 ± 0.007	0.0006±0.0001	0.0008±0.0001
Orotate	0.006±0.002	N.D.	0.04 ± 0.001	0.005 ± 0.0008
Acetate	0.71 ± 0.09	0.56 ± 0.03	0.67 ± 0.09	0.62 ± 0.06
Fumarate	0.0022 ± 0.0012	0.008 ± 0.001	0.004±0.001	0.002±0.001
Succinate	0.14 ± 0.036	N.D.	0.16 ± 0.04	0.15 ± 0.02
Ethanol	0.65 ± 0.11	0.61 ± 0.08	0.77 ± 0.04	0.46 ± 0.08

The table indicates specific growth rates during the exponential phase of growth given in doublings per hour. Product yields are indicated in mol product produced per mol of glucose consumed. N.D. in yields meana that the specific product could not be detected throughout the time course of the measurements. Data are average values of at least three biological replicates.

Furthermore, AV34 excretes dihydroorotate (DHO) instead of orotate. Both metabolites are precursors in pyrimidine synthesis. The DHO dehydrogenase is known to transfer electrons from DHO to quinone [44, 45]. Obviously, this step is inhibited in the mutant AV34 lacking MK and DMK under anaerobic conditions.

### Quinone distribution and ArcA phosphorylation

**Fig 2:**
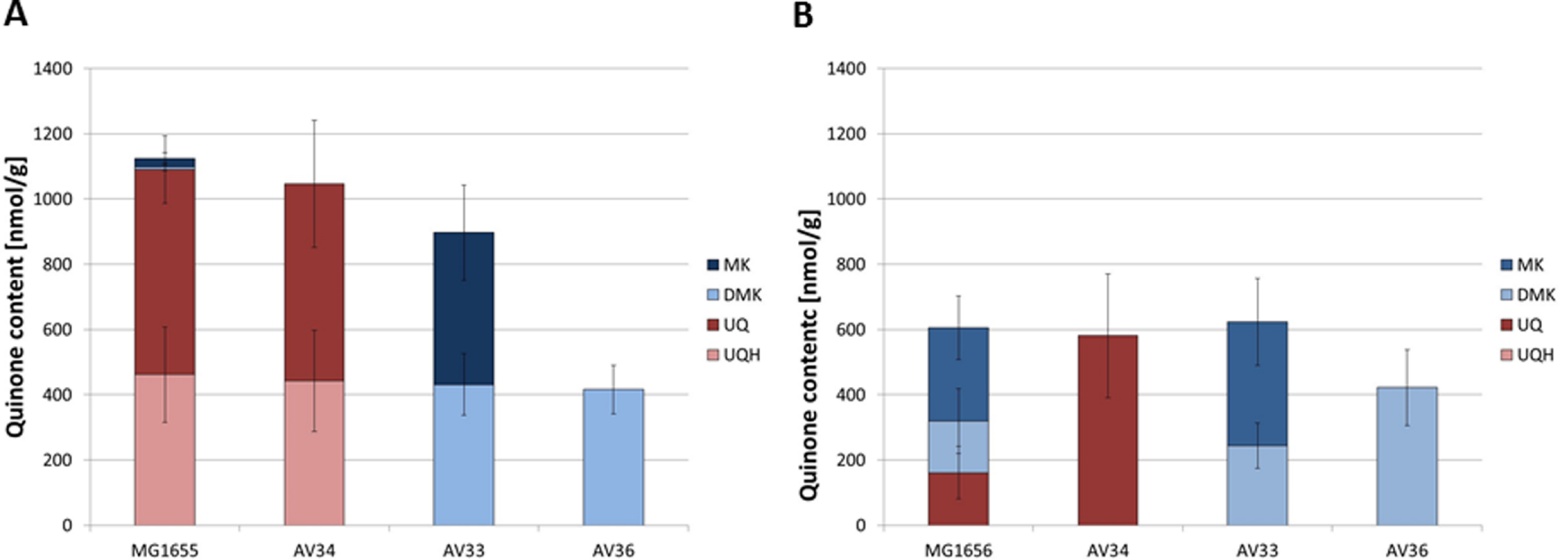
**Quinone content of the wild type MG1655 and the mutants during exponential growth under aerobic (A) and anaerobic (B) batch conditions.** The values represent the average values from at least three independent experiments.

Changes in the redox state and the composition of the quinone pool during an aerobic-anaerobic transition were observed previously [26]. To analyze how defects in the synthesis of one or two quinone species influence the amount of the remaining quinones, we compared the quinone content of the three mutants to that of the wild type MG1655 (Fig 2). Similar studies have already been published [31, 38], but since the growth properties of the mutants determined by us deviate from those observed by Sharma and coworkers, we also repeated the analysis of the quinone content. In contrast to van Beilen and coworkers [38] who analyzed ArcA phosphorylation during shifts in aeration, we were careful to analyze exponential growth under constant aerobiosis conditions. A direct comparision of the data is hence not possible.

Under aerobic conditions, the total UQ content in MG1655 amounted to 1090 nmol/g_DCW,_ which was about 7 fold higher than under anaerobic conditions. Notably, under aerobic conditions, about 40% of the quinone pool was in the reduced state, while no reduced UQ was detected under anaerobic conditions. This is contrary to expectation. The amounts of DMK and MK in MG1655 were about 9 fold higher under anaerobic than under aerobic conditions. Under aerobic conditions, AV34 showed a similar UQ amount as MG1655. In contrast during fermentative growth, the UQ content in AV34 was significantly higher than in MG1655 and only oxidized UQ was detected. The strains AV33 and AV36 showed significantly higher amounts of MK and/or DMK under aerobic conditions than MG1655. For completeness, the relative composition of the quinone pool is listed in Table 5. Summarizing, the absence of one quinone species leads to an increase of the amount of the remaining species while the differences between aerobic and anaerobic conditions are less pronounced.

**Table 5:** Relative ArcA phosphorylation and quinone distribution under aerobic (+O_2_) and anaerobic (-O_2_) conditions.

	MG1655_(UQ,DMK,MK)_		AV34 _(UQ)_		AV33 _(DMK,MK)_		AV36 _(MK)_	
	+ O_2_	- O_2_	+ O_2_	- O_2_	+ O_2_	- O_2_	+ O_2_	- O_2_
ArcA~P [%]	3 ± 3	54 ± 9	9 ± 7	56 ± 10	4 ± 2	19 ± 7	6 ± 4	29± 7
Ratio [%]								
UQH	41 ± 10	N.D.	42 ± 15	N.D.	N.D.	N.D.	N.D.	N.D.
UQ	56 ± 9	27 ± 13	58 ± 19	100 ± 0	N.D.	N.D.	N.D.	
DMK	0.68 ± 1	26 ± 16	N.D.	N.D.	48 ± 11	39 ± 11	100 ± 0	100 ± 0
MK	2.24 ± 2	47 ± 16	N.D.	N.D.	52 ± 16	61 ± 21	N.D.	N.D:

The table summarizes the data for ArcA phosphorylation determined by Phos-tag gel electrophoresis as well as the composition of the quinone pool during aerobic and anaerobic growth, respectively. All values were calculated from samples of at least three independet growth curves. N.D. means that no signal was detected for this species in HPLC.

Quinones have been implicated in the control of ArcB kinase activity but different studies came to different conclusions about the type of quinone responsible for ArcB activation or deactivation [16, 31, 38]. We hence checked ArcA phosphorylation in the different strains by Phostag™ SDS PAGE and Western blotting. An example image of a Western Blot is shown in Fig C in S1 File.

As expected, the wild type showed only low ArcA phosphorylation of about 5% during aerobic growth (Table 5) [29, 32, 38]. No significant differences were observed for the different mutant strains. Under anaerobic conditions the picture was more diverse. The wild type and AV34 showed about 50% phosphorylation, confirming previous studies [29, 31, 46]. In contrast, ArcA phosphorylation of AV33 and AV36, the mutants lacking UQ, was reduced compared to the wild type. Based on our data, it cannot be decided if the lack of a quinone species or the changes in growth and product synthesis are responsible for the observed differences in ArcA phosphorylation.

### Aerobic and anaerobic gene expression analysis

**Fig 3:**
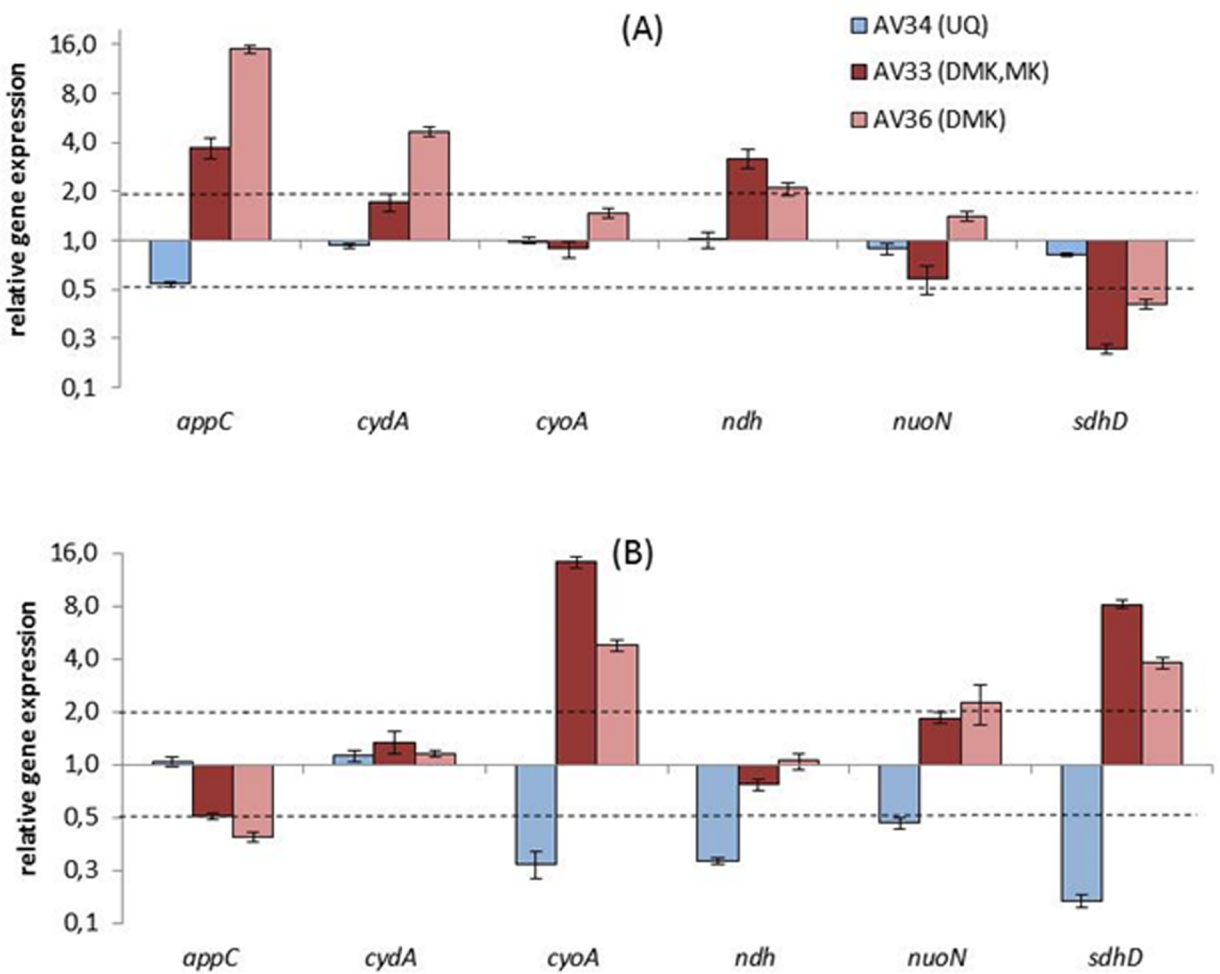
**Aerobic (A) and anaerobic (B) gene expression analysis**. Genes encoding the terminal oxidases cytochrome bd II (*appC*), cytochrome bd (*cydA*) and cytochrome bo_3_ (*cyoA*) and dehydrogenases NADH: quinone oxidoreductase II (*ndh*), NADH: ubiquinone oxidoreductase (*nuoN*) and succinate dehydrogenase (*sdhD)* were analysed. The transcription pattern was normalized to the reference genes *recA* and *rpoD* and to the expression of the wild type strain MG1655 under aerobic (A) and anaerobic (B) conditions respectively. Due to the normalization, constant or unchanged relative gene expression levels are calculated as 1. The Y-axis was formatted in logarithmic scale with base 2, to equally visualize up and downregulation of genes.

In order to analyze the adaptation of the central metabolism in the mutants strains to aerobic and anaerobic growth, gene expression analyses of important genes related to the TCA cycle, respiratory chain and mixed acid fermentation were conducted via qRT-PCR (Fig 3) (Fig E and Table A in S1 File). Fig 3 shows the relative expression levels for selected genes from this study. Data for all genes analyzed are shown in Table A in S1 File. The general picture of gene expression for aerobic and anaerobic conditions is not changed in the mutant strains compared to MG1655 and is in agreement with literature data [Fig E in S1 File] [4, 5, 32, 47, 48]. As expected, genes connected to TCA cycle or respiration were downregulated during anaerobic growth [Fig E in S1 File]. The extent of changes varies between the mutants though. Under aerobic conditions, the UQ mutants AV33 and AV36 showed an increased expression of *appC*, *cydA* and *ndh*. This means that these genes, which are active under microaerobic to anaerobic conditions, are less strongly downregulated under aerobic conditions in AV33 and AV36 than in MG1655. AV33 and AV36 also showed a slight downregulation of *sdhD* under aerobic conditions, compared to MG1655 and AV34. This fits to their slow growth and increased lactate production under aerobic conditions, hinting to a less active TCA cycle. As ArcA phosphorylation does not change under these conditions, the observed changes in gene expression cannot be attributed to ArcA regulation.

Under anaerobic conditions, growth of AV33 and AV36 did not change, but there was still an obvious change in gene expression levels of a number of genes. In AV33 and to a lesser extent in AV36 *cyoA*, *sdhD* and *nuoN* were upregulated during anaerobic growth. This change in gene expression reflects the observed lower ArcA phosphorylation levels in AV33 and AV36. Notably, *appC* was upregulated during aerobic growth in AV33 and AV36, while it is slightly downregulated in these strains under anaerobic conditions. For AV34 with only UQ a low but significant downregulation of *cyoA*, *ndh*, and *sdhD* was observed.

## Discussion

The facultative anaerobic microorganism *E*. *coli* is able to grow in the presence as well as in the absence of oxygen or alternative electron acceptors. To be able to adapt to variable surrounding conditions a complex network of alternative reactions is present. The adaption to the presence or absence of oxygen is guided by two main regulators, the repressor protein FNR and the two-component system ArcB/A, that repress or activate various genes related to respiration and fermentation.

In this study we characterized a set of mutants of *E*. *coli* MG1655 that are able to synthesize only one or two of the quinone species. These mutants were analyzed with respect to growth, product synthesis and gene expression. In addition, the phosphorylation state of ArcA was determined directly using Phos-tag gel electrophoresis coupled to Western Blotting and the expression of selected genes was determined by qRT-PCR. The goal was to get a complete picture of the changes in a cell’s physiology provoked by mutations in quinone synthesis. Unlike in previous studies [38], we focused on batch growth under either aerobic or fermentative conditions.

Under aerobic conditions, strains AV33 and AV36, which both lack UQ, show a considerably lower growth rate [Table 3]. This is coupled with the production of lactate. Obviously, the deletion of UQ, the main quinone under aerobic conditions, prevents the efficient regeneration of NADH via the electron transport chain, leading to a (partial) fermentative behavior [1, 39]. Previously it was reported that a Δ*ubiE* mutant shows a lower oxygen uptake than the wild type [49], confirming our observation. It is known that the succinate dehydrogenase complex (SDH) functions with UQ as electron acceptor [23]. Due to the lack of UQ both, the electron transport chain and the SDH reaction, are impaired, resulting in the accumulation of NADH and possibly also in an accumulation of metabolites of the pyruvate:acetyl-CoA node. In this case NADH is regenerated by the production of lactate from pyruvate and a further accumulation of pyruvate is prevented. Notably, the expression of *sdhD*, which is part of the operon encoding SDH components, is slightly downregulated in AV33 and AV36 compared to MG1655. This downregulation might be explained by global changes e.g. of cAMP due to the lower growth rate. It cannot be explained, however, by changes in the ArcA phosphorylation level, which does not change in the different mutants under aerobic growth conditions. Also, no changes in the FNR activity are to be expected as the external conditions are constant. The growth rates obtained differ from those determined in the study of Sharma and coworkers [31]. AV34 shows a similar aerobic growth rate as MG1655 in our hands while its growth rate was significantly decreased in the study of Sharma and coworkers [31]. As the same mutant was used in both studies, the differences are hard to explain. At the same time the growth rates of AV33 and AV36 are more reduced in our hands than by Sharma and coworkers [31]. This effect might be explained by the addition of 1% LB to the growth medium by Sharma and coworkers.

Notably, it was observed that AV36, which can synthesize DMK only, grows slightly faster and excretes less lactate under aerobic conditions than AV33, which can still synthesize DMK and MK. Also, downregulation of *sdhD* is less pronounced in AV36. This indicates an inhibitory effect of high amounts of MK if present under aerobic conditions. Our analysis indicates an increased production of superoxide anions in AV33 and AV36, with AV33 showing higher oxidative stress. This might be explained by the increased amounts of MK and DMK present in these strains under aerobic conditions, as well as by the lack of ubiquinone that is known to protect cells from oxidative stress [40]. MK does not function optimally under aerobic conditions due to a high non-catalytic oxidation rate [50]. Also the MK analogue menadione can be used to induce oxidative stress in *E*. *coli* [21]. It is conceivable that strains expressing increased amounts of MK or DMK in the presence of oxygen suffer from oxidative stress. This might be an additional reason for the low growth rate of these strains and could explain why AV33 grows even worse under aerobic conditions. In line with these observations, a frequent occurrence of secondary mutations in a mutant producing MK only under aerobic conditions was reported [38]. AV36 excretes less lactate than AV33. This indicates that DMK is able to partially replace UQ in the aerobic respiration chain, while MK is not. In AV33 and AV36 considerably higher levels of MK and DMK than in the wild type strain can be detected during aerobic growth. Obviously, the lack of UQ is counterbalanced by an increased production of MK and DMK. The competition of the UQ and MK precursors for the same methylase, UbiE is a potential reason for this shift [51].

Under anaerobic conditions the picture is more diverse. Strain AV34, which can only synthesize UQ, is characterized by a significant growth defect under anaerobic conditions, while the UQ deletion strains AV33 and AV36 grow only slightly slower than the wild type. AV34 is not able to produce succinate [Tab.4] which could be expected because fumarate reductase functions with MK as electron donor [23]. Also, it was observed that AV34 excretes dihydroorotate instead of orotate. Orotate has been regarded as an overflow metabolite in addition to acetate [52]. The production of dihydroorotate in AV34 indicates that the enzyme dihydroorotate dehydrogenase requires MK or DMK as electron acceptor under anaerobic conditions [53] and is not able to function efficiently with UQ. Orotate is a precursor of pyrimidine nucleotides. The impairment of this reaction hence might contribute to the very slow growth of AV34. Furthermore, AV34 shows a decreased formation of formate as well as a slightly higher excretion of pyruvate under anaerobic conditions. The decreased amount of formate, which is not coupled to a decreased production of other products of the PFL, might reflect a higher activity of FHL, an assumption that is further confirmed by the profiles of H_2_ and CO_2_ measured during fermentative growth [Fig B in S1 File]. There are no reports about quinones being active in the control of FHL activity or expression so far. Alternatively, it cannot be ruled out that the observed differences in the fermentation products in this mutant are due to a slightly active pyruvate dehydrogenase complex also during anaerobic growth as previously discussed [54–57]

While in spite of its slow growth the UQ only mutant, AV34, mirrored the high ArcA phosphorylation level (about 50%) of the wild type under anaerobic conditions, the mutants lacking UQ show a reduced ArcA phosphorylation. It has been reported that UQ inhibits ArcB autophosphorylation and hence ArcA phosphorylation while MK is believed to enhance ArcB phosphorylation [13, 15, 38]. This is contrary to our results that show an unchanged phosphorylation level in the UQ only strain and a reduced level in the strains lacking UQ. Our data are not sufficient to explain this discrepancy and the reason for this can only be speculated. It is tempting to assume that there are significant differences between short and long term responses to changes in aerobiosis. While during shifts in oxygen supply the observed inhibitory effect of UQ on ArcA phosphorylation is visible [16, 38], it might be overridden by additional control mechanisms and adaptations at later stages and during continued growth under a given oxygen concentration. The concentrations of the quinone species might change with adaption to growth under aerobic or anaerobic conditions, respectively. So far, the published data for the concentrations of the different quinone species during an aerobe-anaerobe shift or vice versa cover only a time span of about 1–2 hours [29, 38]. The concentration of the quinone species is, besides their redox potential, one additional factor that determines the ability to accept or donate electrons from/to the sulfur groups of ArcB [38]. Also, the concentration of ArcB and/or ArcA might vary although no regulation of *arcB* or *arcA* expression has been reported. Furthermore, it has been shown that metabolites e.g. D-lactate which accumulate during growth influence the ArcA activity [30, 58, 59].

Under aerobic conditions, some ArcA controlled genes showed differences in gene expression in spite of almost complete ArcA dephosphorylation in all strains. Either in this case further regulators e.g. cAMP-CRP are differentially active in the quinone mutants or ArcA activity can also be controlled independently of phosphorylation. Metabolic flux ratio analysis indicates that the knockout of ArcA influences the flux through the TCA cycle while an ArcB knockout had no effect [60]. As expression of many genes varies strongly in AV34 while changes in AV33 to AV36 are only small, it is tempting to speculate that quinones might be involved in this possible phosphorylation independent control by ArcA.

In summary, our data show that the interplay of the three quinone species in *E*. *coli* is necessary to allow optimal growth under aerobic and also anaerobic batch conditions. They confirm previous studies showing that UQ is the main quinone under aerobic conditions and show that DMK and MK are not only abundant during anaerobic respiration but also during fermentation. An important function of DMK and MK under fermentative conditions can be reasoned from the strongly impaired fermentative growth of an UQ only mutant. On the other hand, mutants lacking UQ are not able to respire efficiently. MK even has an inhibitory effect on growth while DMK is able to replace UQ at least in part in aerobic respiration. Notably, the lack of UQ is not affecting ArcA phosphorylation during aerobic batch growth. Mutants lacking UQ show an almost normal fermentative growth phenotype. Strikingly, these mutants display a lower ArcA phosphorylation and also small variations in gene expression but these changes don't seem to impact growth.

## Supporting information

S1 File **Fig A:** Mean fluorescence of cells incubated with CellRox Green. Shown are the mean fluorescence values of the different strains after addition of 100 μM menadione to the aerobically growing cultures about 1 h before incubation with Cellrox Green. **Fig B**: Comparison of growth and H_2_ and CO_2_ production of AV34 and MG1655 under fermentative conditions. Biomass measurements as well as concentrations of H_2_ and CO_2_ in the offgas are plotted. As can be seen from A) AV34 produces significant amounts of H_2_ and CO_2_ during the exponential growth phase. For unknown reasons production increases at about 15 h in midexponential phase. B) MG1655 shows a different behavior. Here H_2_ and CO_2_ production start in late exponential phase. **Fig C:** ArcA phosphorylation under anaerobic conditions. Western Blot of a Phos-tag gel with samples of MG1655 and the quinone mutants grown under anaerobic conditions to analyze the relative ArcA Phosphorylation in vivo. **Fig D**: D-lactate formation of quinone mutants compared to MG1655 under anaerobic batch condition. Shown are time course data for lactose concentrations measured during growth experiments. **Fig E**: Gene expression analysis of MG1655 and the quinone mutants under anaerobic batch conditions. Data normalized on MG1655 under aerobic batch conditions. Due to the normalization, constant or unchanged relative gene expression levels are calculated as 1. The Y-axis was formatted in logarithmic scale with base 2, to equally visualize up and downregulation of genes.
(DOCX)

S1 Dataset This file contains the following: **Suppl_data_aerobe.docx**: Time course data for biomass and by-products from aerobic growth experiments. **Suppl_data_anaerobe.docx**: Time course data for biomass and by-products from anaerobic growth experiments. **Suppl_data_CellRox**: Individual data from oxidative stress measurements. **Suppl_data_Quinone_aerobe.docx**: Individual quinone concentrations from aerobic growth experiments. **Suppl_data_Quinone_anaerobe.docx**: Individual quinone concentrations from anaerobic growth experiments.
(ZIP)
