# Supplementary material for: All three quinone species play distinct roles in ensuring optimal growth under aerobic and fermentative conditions in E. coli K12
Source: PLoS One. 2018 Apr 3;13(4):e0194699. doi: 10.1371/journal.pone.0194699 (PMC5882134; doi:10.1371/journal.pone.0194699)
Supplement: S1 File — Fig A: Mean fluorescence of cells incubated with CellRox Green. Shown are the mean fluorescence values of the different strains after addition of 100 μM menadione to the aerobically growing cultures about 1 h before incubation with Cellrox Green. Fig B: Comparison of growth and H2 and CO2 production of AV34 and MG1655 under fermentative conditions. Biomass measurements as well as concentrations of H2 and CO2 in the offgas are plotted. As can be seen from A) AV34 produces significant amounts of H2 and CO2 during the exponential growth phase. For unknown reasons production increases at about 15 h in midexponential phase. B) MG1655 shows a different behavior. Here H2 and CO2 production start in late exponential phase. Fig C: ArcA phosphorylation under anaerobic conditions. Western Blot of a Phos-tag gel with samples of MG1655 and the quinone mutants grown under anaerobic conditions to analyze the relative ArcA Phosphorylation in vivo. Fig D: D-lactate formation of quinone mutants compared to MG1655 under anaerobic batch condition. Shown are time course data for lactose concentrations measured during growth experiments. Fig E: Gene expression analysis of MG1655 and the quinone mutants under anaerobic batch conditions. Data normalized on MG1655 under aerobic batch conditions. Due to the normalization, constant or unchanged relative gene expression levels are calculated as 1. The Y-axis was formatted in logarithmic scale with base 2, to equally visualize up and downregulation of genes. (DOCX) [file pone.0194699.s001.docx]

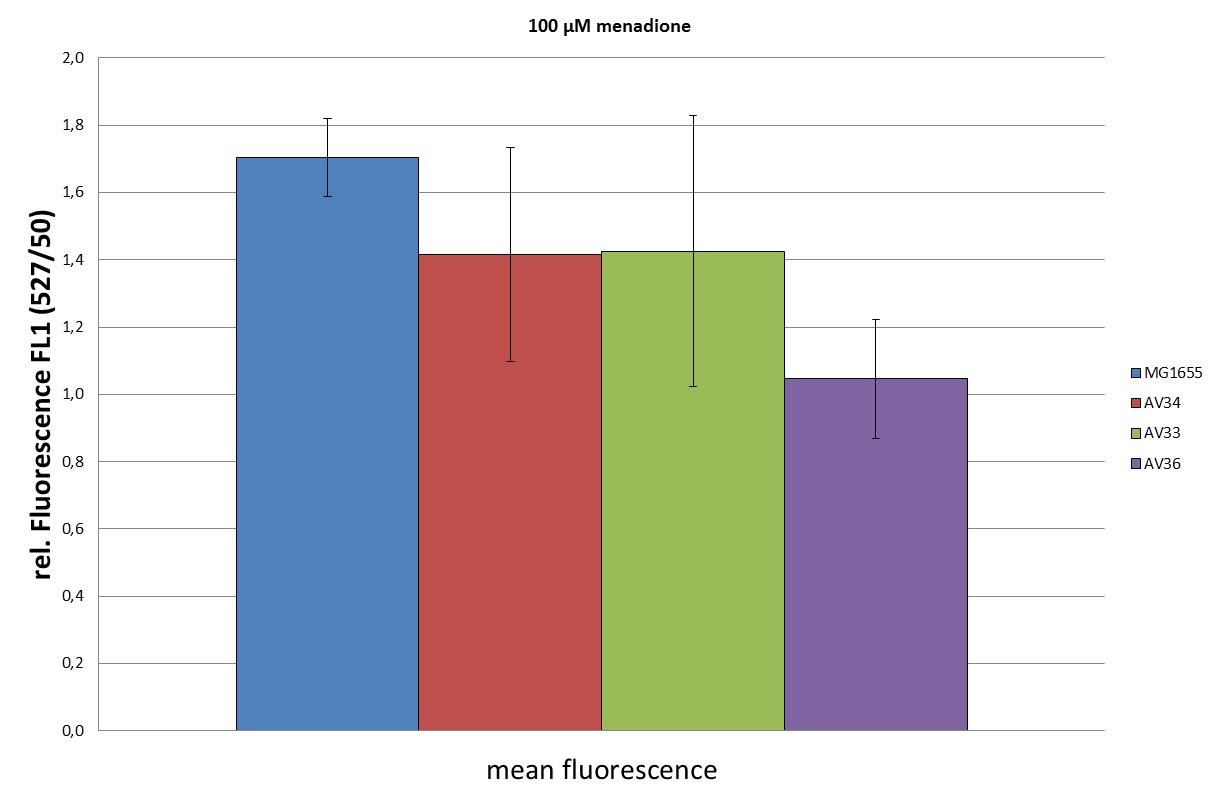


**Fig A: Mean fluorescence of cells incubated with CellRox Green.** Shown are the mean fluorescence values of the different strains after addition of 100 µM menadione to the aerobically growing cultures about 1 h before incubation with Cellrox Green

##
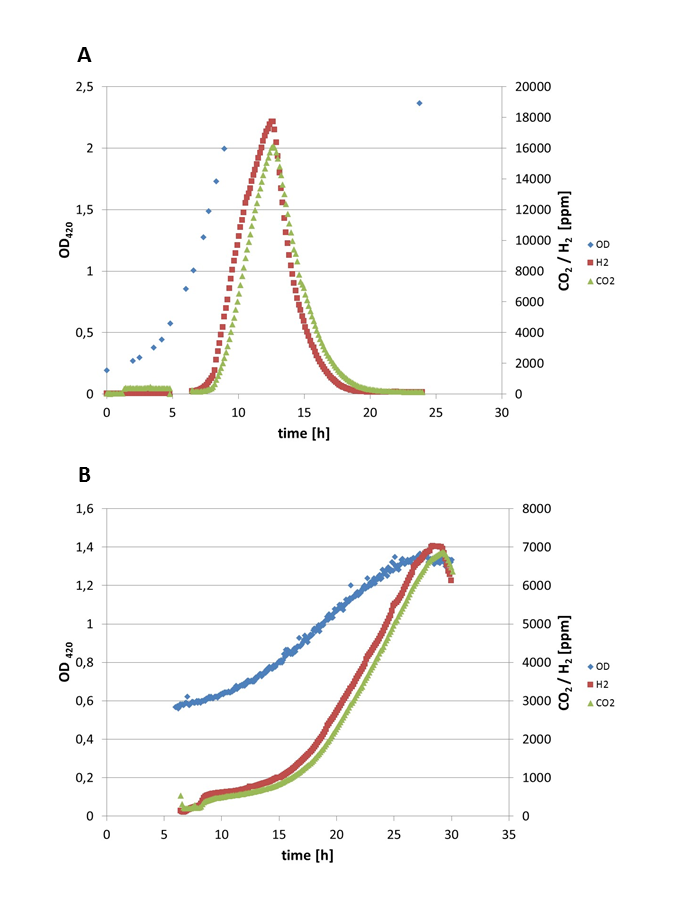


**Fig B**: **Comparison of growth and H_2_ and CO_2_ production of AV34 and MG1655 under fermentative conditions**. Biomass measurements as well as concentrations of H_2_ and CO_2_ in the offgas are plotted. As can be seen from A) AV34 produces significant amounts of H_2_ and CO_2_ during the exponential growth phase. For unknown reasons production increases at about 15 h in midexponential phase. B) MG1655 shows a different behavior. Here H_2_ and CO_2_ production start in late exponential phase

Experiments were performed in an Infors bioreactor with 400 ml culture volume. To assure anaerobic conditions, cultures were gassed with 10 ml/min pure N_2_. Biomass of AV34 was determined via a biomass probe (Optek), to be able to monitor the long growth phase without gaps. H_2_ and CO_2_ concentrations in the off-gas were monitored by an Agilent Micro-GC CP 490 equipped with columns Molecular Sieve MS5A, 10m and Poraplot PPU, 10m.

**Std aerobic**

**MG1655**

**AV36**

**Std anaerobic**

**AV33**

**AV34**

**Marker**


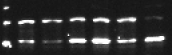


**<- ArcA-P**

**<- ArcA**

**MG1655**

Fig C. ArcA phosphorylation under anaerobic conditions. Western Blot of a Phos-tag gel with samples of MG1655 and the quinone mutants grown under anaerobic conditions to analyze the relative ArcA Phosphorylation in vivo.

Fig D. D-lactate formation of quinone mutants compared to MG1655 under anaerobic batch condition

Fig E: Gene expression analysis of MG1655 and the quinone mutants under anaerobic batch conditions. Data normalized on MG1655 under aerobic batch conditions. Due to the normalization, constant or unchanged relative gene expression levels are calculated as 1. The Y-axis was formatted in logarithmic scale with base 2, to equally visualize up and downregulation of genes.

Table A. Summary of aerobic and anaerobic gene expression data of the quinone mutants. The transcription pattern was normalized to the reference genes *recA* and *rpoD* and to the expression of the wild-type strain MG1655. In addition, the transcription factors that inhibit (red) or activate (green) expression of these genes according to [www.ecocyc.com](http://www.ecocyc.com) are indicated.

|  |  | **Aerobic** | | | **Anaerobic** | | |
| --- | --- | --- | --- | --- | --- | --- | --- |
| *Gene* | Transcription factors | **AV34** | **AV33** | **AV36** | **AV34** | **AV33** | **AV36** |
| *Quinone present* |  | UQ | DMK/MK | DMK | UQ | DMK/MK | DMK |
| *ArcA-P [%]* |  | 9 | 4 | 6 | 56 | 19 | 29 |
| *adhE* | Fis, FNR, Cra, Lrp, NarL | 1.10 ±0.05 | 5.33 ±0.21 | 3.20 ±0.15 | 0.63 ±0.01 | 1.11 ±0.03 | 1.15 ±0.04 |
|  |  |  |  |  |  |  |  |
| *frdA* | DcuR P, FNR, NarL P | 1.17 ±0.08 | 1.71 ±0.06 | 1.53 ±0.04 | 1.79 ±0.07 | 0.91 ±0.03 | 0.93 ±0.03 |
|  |  |  |  |  |  |  |  |
| *ldhA* |  | 0.74 ±0.05 | 1.54 ±0.06 | 5.21 ±0.62 | 1.61 ±0.03 | 0.50 ±0.01 | 0.48 ±0.02 |
|  |  |  |  |  |  |  |  |
| *pflB* | ArcA P, cAMP CRP, FNR, IhfB/A, Fis, NarL P | 3.18 ±0.83 | 1.90 ±0.07 | 1.51 ±0.12 | 1.04 ±0.04 | 0.81 ±0.06 | 0.81 ±0.03 |
|  |  |  |  |  |  |  |  |
| *appC* | AppY, ArcA P, YdeO | 0.54 ±0.02 | 3.69 ±0.53 | 14.99 ±0.71 | 1.18 ±0.05 | 0.41 ±0.01 | 0.32 ±0.01 |
|  |  |  |  |  |  |  |  |
| *cydA* | ArcA P, Cra, HypT[Met-oxidized], FNR, H-NS | 0.93 ±0.04 | 1.73 ±0.22 | 4.68 ±0.28 | 1.32 ±0.07 | 1.34 ±0.04 | 1.16 ±0.05 |
|  |  |  |  |  |  |  |  |
| *mdh* | cAMP CRP, DpiA P, ArcA P, FlhC/FlhD | 1.46 ±0.10 | 0.84 ±0.02 | 0.88 ±0.03 | 1.26 ±0.03 | 3.63 ±0.09 | 2.89 ±0.13 |
|  |  |  |  |  |  |  |  |
| *sdhCD* | cAMP CRP Fe²^+^ Fur ArcA P FNR | 0.82 ±0.02 | 0.22 ±0.01 | 0.41 ±0.03 | 0.81 ±0.03 | 10.19 ±0.17 | 6.54 ±0.24 |
|  |  |  |  |  |  |  |  |
| *cyoA* | cAMP CRP CusR P Fis GadE ArcA P Cra FNR PdhR Fur YedW P | 0.99 ±0.04 | 0.89 ±0.10 | 1.47 ±0.10 | 2.72 ±0.05 | 24.11 ±0.64 | 11.79 ±0.38 |
|  |  |  |  |  |  |  |  |
| *ndh* | Fis ArcA P FNR Fur IhfB/A NsrR PdhR | 1.00 ±0.11 | 3.21 ±0.43 | 2.09 ±0.19 | 0.79 ±0.02 | 1.42 ±0.06 | 1.24 ±0.04 |
|  |  |  |  |  |  |  |  |
| *nuoN* | Fis NarL P ArcA P FNR IhfB/A | 0.89 ±0.07 | 0.58 ±0.12 | 1.41 ±0.11 | 0.51 ±0.03 | 1.99 ±0.08 | 1.72 ±0.10 |
|  |  |  |  |  |  |  |  |
| *poxB* | Cra MarA SoxS | 0.34 ±0.02 | 3.74 ±0.46 | 3.83 ±0.53 | 1.17 ±0.02 | 0.77 ±0.03 | 0.77 ±0.03 |
|  |  |  |  |  |  |  |  |
| *carA* | Fis RutR IhfBA ArgR arg PepA PurR |  |  |  | 4.64 ±0.18 | 0.80 ±0.02 | 0.94 ±0.02 |
|  |  |  |  |  |  |  |  |
| *pyrB* | ppGpp |  |  |  | 21.5 ±0.94 | 1.02 ±0.03 | 1.15 ±0.05 |
|  |  |  |  |  |  |  |  |
| *pyrE* |  |  |  |  | 1.04 ±0.03 | 0.62 ±0.03 | 0.63 ±0.02 |
