# Supplementary material for: All three quinone species play distinct roles in ensuring optimal growth under aerobic and fermentative conditions in E. coli K12
Source: PLoS One. 2018 Apr 3;13(4):e0194699. doi: 10.1371/journal.pone.0194699 (PMC5882134; doi:10.1371/journal.pone.0194699)
Supplement: S1 Dataset — This file contains the following: Suppl_data_aerobe.docx: Time course data for biomass and by-products from aerobic growth experiments. Suppl_data_anaerobe.docx: Time course data for biomass and by-products from anaerobic growth experiments. Suppl_data_CellRox: Individual data from oxidative stress measurements. Suppl_data_Quinone_aerobe.docx: Individual quinone concentrations from aerobic growth experiments. Suppl_data_Quinone_anaerobe.docx: Individual quinone concentrations from anaerobic growth experiments. (ZIP) [file pone.0194699.s002.zip › Suppl_Data/Suppl_data_aerobe.docx]

1. Time course data of the different growth curves of MG1655 under aerobic conditions.

| Time [h] | | OD420 | Glc [mM] | Ace [mM] | Lac [mM] | Pyr [mM] |
| --- | --- | --- | --- | --- | --- | --- |
| 0.00 | | 0.3985 | n.d. | n.d. | n.d. | n.d. |
| 1.33 | | 0.694 | 20.33 | 0.41 | 0 | 0 |
| 3.33 | | 2.56 | 13.55 | 2.54 | 0 | 0 |
| 4.42 | | 6.39 | 4.46 | 4.94 | 0 | 0 |
| 8.75 | | 8.89 | 0.07 | 14.76 | 0 | 0 |
| 23.33 | | 7.78 | n.d. | n.d. | n.d. | n.d. |
|  |  | |  |  |  |  |
| Time [h] | OD420 | | Glc [mM] | Ace [mM] | Lac [mM] | Pyr [mM] |
| 0.00 | 0.19 | | 22.96 | nd | 0 | 0 |
| 0.50 | 0.20 | | 22.65 | nd | 0 | 0 |
| 1.00 | 0.22 | | 22.79 | nd | 0 | 0 |
| 1.50 | 0.24 | | 22.03 | nd | 0 | 0 |
| 2.00 | 0.36 | | 22.62 | nd | 0 | 0 |
| 2.50 | 0.53 | | 21.40 | nd | 0 | 0 |
| 3.00 | 0.85 | | 20.89 | nd | 0 | 0 |
| 4.00 | 1.66 | | 18.25 | nd | 0 | 0 |
| 5.00 | 3.53 | | 14.44 | nd | 0 | 0 |
| 6.00 | 6.18 | | 8.47 | nd | 0 | 0 |
| 7.00 | 5.98 | | 5.40 | nd | 0 | 0 |
| 8.00 | 6.28 | | 4.52 | nd | 0 | 0 |
| 9.00 | 6.40 | | 2.91 | nd | 0 | 0 |
| 10.83 | 6.60 | | 1.67 | nd | 0 | 0 |
|  |  | |  |  |  |  |
| Time [h] | OD420 | | Glc [mM] | Ace [mM] | Lac [mM] | Pyr [mM] |
| 0.0 | 0.31 | | n.d. | n.d. | n.d. | n.d. |
| 1.1 | 0.50 | | 21.45 | 0.00 | 0 | 0 |
| 2.0 | 0.89 | | 19.86 | 0.93 | 0 | 0 |
| 2.8 | 1.77 | | 16.09 | 2.09 | 0 | 0 |
| 3.6 | 2.61 | | 14.03 | 2.90 | 0 | 0 |
| 4.1 | 3.29 | | 9.57 | 3.85 | 0 | 0 |
| 4.6 | 4.34 | | 6.34 | 4.89 | 0 | 0 |
| 5.5 | 8.78 | | 0.16 | 4.95 | 0 | 0 |
| 7.2 | 9.45 | | 0.00 | 0.00 | 0 | 0 |
|  |  | |  |  |  |  |

| Time [h] | OD420 | Glc [mM] | Ace [mM] | Lac [mM] | Pyr [mM] |
| --- | --- | --- | --- | --- | --- |
| 0.0 | 0.18 | n.d. | n.d. | n.d. | n.d. |
| 1.0 | 0.28 | 19.33 | 0.32 | 0 | 0 |
| 2.3 | 0.68 | 16.68 | 1.21 | 0 | 0 |
| 3.2 | 1.28 | 16.28 | 3.39 | 0 | 0 |
| 4.3 | 2.99 | 12.44 | 3.51 | 0 | 0 |
| 4.9 | 5.44 | 0.03 | n.d. | n.d. | n.d. |
| 6.8 | 9.15 | 0.00 | 0.00 | 0 | 0 |
| 24.7 | 8.50 | 0.00 | 0.00 | 0 | 0 |
|  |  |  |  |  |  |
| Time [h] | OD420 | Glc [mM] | Ace [mM] | Lac [mM] | Pyr [mM] |
| 0.00 | 0.27 | n.d. | n.d. | n.d. | n.d. |
| 1.00 | 0.38 | 19.20 | nd. | 0 | 0 |
| 2.00 | 0.68 | 19.06 | nd | 0 | 0 |
| 2.75 | 0.96 | 17.74 | nd | 0 | 0 |
| 3.50 | 1.91 | 15.42 | nd | 0 | 0 |
| 3.92 | 2.72 | 13.44 | nd | 0 | 0 |
| 4.25 | 3.53 | 10.87 | nd | 0 | 0 |
| 5.25 | 6.99 | 2.78 | nd | 0 | 0 |
| 7.25 | 7.97 | 0.11 | nd | 0 | 0 |
| 25.75 | 7.57 | 0.01 | nd. | 0 | 0 |
|  |  |  |  |  |  |
| Time [h] | OD420 | Glc [mM] | Ace [mM] | Lac [mM] | Pyr [mM] |
| 0.0 | 0.18 | n.d. | n.d. | n.d. | n.d. |
| 1.0 | 0.24 | n.d. | n.d. | n.d. | n.d. |
| 1.8 | 0.28 | n.d. | n.d. | n.d. | n.d. |
| 3.8 | 1.50 | n.d. | n.d. | n.d. | n.d. |
| 4.5 | 2.65 | n.d. | n.d. | n.d. | n.d. |
| 5.3 | 4.55 | n.d. | n.d. | n.d. | n.d. |
| 6.0 | 7.67 | n.d. | n.d. | n.d. | n.d. |
| 6.5 | 8.40 | n.d. | n.d. | n.d. | n.d. |
| 7.0 | 8.94 | n.d. | n.d. | n.d. | n.d. |

2. Time course data of the different growth curves of AV34 under aerobic conditions.

| Time [h] | OD420 | Glc [mM] | Ace [mM] | Lac [mM] | Pyr [mM] |
| --- | --- | --- | --- | --- | --- |
| 0.00 | 0.20 | n.d. | n.d. | n.d. | n.d. |
| 1.08 | 0.34 | 20.79 | n.d. | 0 | 0 |
| 2.17 | 0.70 | 14.53 | n.d. | 0 | 0 |
| 2.83 | 1.28 | n.d. | n.d. | 0 | 0 |
| 4.25 | 4.33 | n.d. | n.d. | 0 | 0 |
| 5.83 | 7.29 | 0.43 | n.d. | 0 | 0 |
| 22.33 | 7.31 | 0.02 | n.d. | 0 | 0 |
|  |  |  |  |  |  |
| Time [h] | OD420 | Glc [mM] | Ace [mM] | Lac [mM] | Pyr [mM] |
| 0.00 | 0.26 | n.d. | n.d. | n.d. | n.d. |
| 1.00 | 0.31 | 20.26 | 0.06 | 0 | 0 |
| 2.00 | 0.49 | 19.33 | 0.31 | 0 | 0 |
| 2.75 | 0.84 | 19.46 | 0.58 | 0 | 0 |
| 3.50 | 1.22 | 15.81 | 0.90 | 0 | 0 |
| 3.92 | 1.77 | 11.14 | 0.60 | 0 | 0 |
| 4.25 | 2.41 | 13.17 | 0.74 | 0 | 0 |
| 5.25 | 4.87 | 7.06 | 1.37 | 0 | 0 |
| 7.25 | 5.95 | 0.26 | 0.00 | 0 | 0 |
| 25.75 | 6.70 | 0.01 | 0.00 | 0 | 0 |
|  |  |  |  |  |  |
| Time [h] | OD420 | Glc [mM] | Ace [mM] | Lac [mM] | Pyr [mM] |
| 0.00 | 0.16 | n.d. | n.d. | n.d. | n.d. |
| 1.58 | 0.26 | 19.45 | 0.25 | 0 | 0 |
| 2.75 | 0.48 | 19.45 | 0.62 | 0 | 0 |
| 4.00 | 1.15 | 16.02 | 1.25 | 0 | 0 |
| 5.25 | 2.29 | 11.85 | 2.38 | 0 | 0 |
| 6.50 | 6.42 | 2.57 | 3.62 | 0 | 0 |
| 7.25 | 7.28 | -0.12 | 0.85 | 0 | 0 |
| 8.08 | 7.96 | -0.11 | 0.00 | 0 | 0 |
| 24.75 | 7.16 | -0.10 | 0.25 | 0 | 0 |
|  |  |  |  |  |  |
| Time [h] | OD420 | Glc [mM] | Ace [mM] | Lac [mM] | Pyr [mM] |
| 0.00 | 0.18 | 22.37 | 0.00 | 0 | 0 |
| 1.00 | 0.22 | 21.45 | 0.00 | 0 | 0 |
| 2.00 | 0.44 | 21.18 | 0.42 | 0 | 0 |
| 3.00 | 0.86 | 19.53 | 0.39 | 0 | 0 |
| 3.67 | 1.46 | 17.01 | 1.48 | 0 | 0 |
| 4.25 | 2.41 | 15.56 | 2.35 | 0 | 0 |
| 4.67 | 3.37 | 11.85 | 3.04 | 0 | 0 |
| 5.08 | 4.45 | 8.67 | 3.55 | 0 | 0 |
| 5.67 | 6.52 | 3.61 | 4.41 | 0 | 0 |
| 6.08 | 8.11 | 0.03 | 4.44 | 0 | 0 |
| 8.58 | 8.09 | 0.07 | 0.48 | 0 | 0 |
|  |  |  |  |  |  |
| Time [h] | OD420 | Glc [mM] | Ace [mM] | Lac [mM] | Pyr [mM] |
| 0.00 | 0.18 | n.d. | n.d. | n.d. | n.d. |
| 1.00 | 0.22 | n.d. | n.d. | n.d. | n.d. |
| 1.75 | 0.25 | n.d. | n.d. | n.d. | n.d. |
| 3.75 | 1.44 | n.d. | n.d. | n.d. | n.d. |
| 4.50 | 2.57 | n.d. | n.d. | n.d. | n.d. |
| 5.25 | 4.55 | n.d. | n.d. | n.d. | n.d. |
| 6.00 | 6.87 | n.d. | n.d. | n.d. | n.d. |
| 6.50 | 7.51 | n.d. | n.d. | n.d. | n.d. |
| 7.00 | 7.35 | n.d. | n.d. | n.d. | n.d. |
| 26.00 | 7.20 | n.d. | n.d. | n.d. | n.d. |

3. Time course data of the different growth curves of AV33 under aerobic conditions.

| Time [h] | OD420 | Glc [mM] | Ace [mM] | Lac [mM] | Pyr [mM] |
| --- | --- | --- | --- | --- | --- |
| 0.00 | 0.38 | n.d. | n.d. | n.d. | n.d. |
| 1.33 | 0.56 | 20.21 | 0.41 | 1.97 | 0.03 |
| 3.33 | 0.76 | 18.19 | 2.54 | 5.13 | 0.02 |
| 4.42 | 0.90 | 14.27 | 4.94 | 8.31 | 0.03 |
| 8.75 | 2.03 | 0.20 | 14.76 | 29.88 | 0.29 |
| 23.33 | 2.46 | 0.01 | n.d. | 22.11 | 0.01 |
|  |  |  |  |  |  |
| Time [h] | OD420 | Glc [mM] | Ace [mM] | Lac [mM] | Pyr [mM] |
| 0.00 | 0.16 | 19.06 | 0.00 | 0.00 | 0.00 |
| 1.67 | 0.24 | 18.40 | 0.00 | 1.18 | 0.00 |
| 2.50 | 0.29 | 18.67 | 0.00 | 2.05 | 0.00 |
| 3.17 | 0.34 | 17.61 | 0.00 | 2.90 | 0.00 |
| 3.92 | 0.40 | 17.81 | 0.00 | 4.02 | 0.04 |
| 6.00 | 0.60 | 14.40 | 0.00 | 8.43 | 0.10 |
| 7.00 | 0.67 | 12.41 | 0.00 | 11.89 | 0.16 |
| 23.83 | 1.13 | 0.03 | 6.23 | 30.30 | 0.05 |
|  |  |  |  |  |  |
| Time [h] | OD420 | Glc [mM] | Ace [mM] | Lac [mM] | Pyr [mM] |
| 0.00 | 0.16 | n.d. | n.d. | n.d. | n.d. |
| 1.00 | 0.21 | 13.77 | 0.31 | 0.63 | 0.02 |
| 2.25 | 0.29 | 14.43 | 0.31 | 1.64 | 0.04 |
| 3.17 | 0.37 | 12.71 | 0.40 | 2.81 | 0.05 |
| 4.25 | 0.48 | 13.11 | 0.50 | 4.72 | 0.09 |
| 6.83 | 0.96 | 10.66 | 1.10 | 11.73 | 0.25 |
| 7.75 | 1.28 | 0.02 | 9.59 | 24.00 | 0.00 |
| 24.67 | 1.96 | n.d. | n.d. | n.d. | n.d. |
|  |  |  |  |  |  |
| Time [h] | OD420 | Glc [mM] | Ace [mM] | Lac [mM] | Pyr [mM] |
| 0.00 | 0.09 | n.d. | n.d. | n.d. | n.d. |
| 1.00 | 0.11 | n.d. | n.d. | n.d. | n.d. |
| 1.75 | 0.15 | n.d. | n.d. | n.d. | n.d. |
| 2.83 | 0.20 | n.d. | n.d. | n.d. | n.d. |
| 3.50 | 0.25 | n.d. | n.d. | n.d. | n.d. |
| 4.17 | 0.25 | n.d. | n.d. | n.d. | n.d. |
| 4.75 | 0.29 | n.d. | n.d. | n.d. | n.d. |
| 7.33 | 0.53 | n.d. | n.d. | n.d. | n.d. |

4. Time course data of the different growth curves of AV36 under aerobic conditions.

| Time [h] | OD420 | Glc [mM] | Ace [mM] | Lac [mM] | Pyr [mM] |
| --- | --- | --- | --- | --- | --- |
| 0.00 | 0.19 | 21.87 | 0.00 | 0.00 | 0.00 |
| 1.00 | 0.25 | 23.51 | 0.00 | 1.16 | 0.00 |
| 1.75 | 0.38 | 20.50 | 0.00 | 2.73 | 0.04 |
| 2.83 | 0.65 | 15.92 | 0.94 | 3.88 | 0.06 |
| 3.50 | 0.67 | 11.04 | 1.21 | 5.17 | 0.09 |
| 4.17 | 0.76 | 6.26 | 1.63 | 7.20 | 0.13 |
| 4.75 | 1.00 | 3.23 | 5.24 | 21.51 | 0.61 |
| 7.33 | 2.85 | 0.18 | n.d. | n.d. | n.d. |
|  |  |  |  |  |  |
| Time [h] | OD420 | Glc [mM] | Ace [mM] | Lac [mM] | Pyr [mM] |
| 0.00 | 0.14 | n.d. | n.d. | n.d. | n.d. |
| 1.58 | 0.19 | 18.08 | 0 | 0.67 | 0.00 |
| 2.75 | 0.30 | 19.72 | 0 | 1.32 | 0.01 |
| 4.00 | 0.50 | 16.71 | 0.31 | 2.61 | 0.09 |
| 5.25 | 0.90 | 14.04 | 0.84 | 5.32 | 0.12 |
| 6.50 | 1.63 | 10.10 | 1.37 | 10.10 | 0.15 |
| 6.75 | 1.89 | n.d. | n.d. | n.d. | n.d. |
| 7.25 | 2.38 | 6.08 | 6.19 | 12.10 | 0.40 |
| 8.08 | 3.15 | 3.05 | 6.25 | 13.49 | 0.61 |
| 24.75 | 2.96 | -0.01 | n.d. | 9.06 | 0 |
|  |  |  |  |  |  |
| Time [h] | OD420 | Glc [mM] | Ace [mM] | Lac [mM] | Pyr [mM] |
| 0.00 | 0.17 | 18.27 | 0.00 | 0.00 | 0.00 |
| 1.67 | 0.29 | 19.06 | 0.00 | 0.79 | 0.00 |
| 2.50 | 0.39 | 18.27 | 0.00 | 1.66 | 0.00 |
| 3.17 | 0.50 | 17.67 | 0.00 | 2.61 | 0.00 |
| 3.92 | 0.62 | 16.81 | 1.33 | 3.93 | 0.07 |
| 6.00 | 1.46 | 10.66 | 3.26 | 10.88 | 0.22 |
| 7.00 | 2.30 | 6.12 | 4.49 | 16.66 | 0.40 |
| 23.83 | 3.26 | -0.03 | 15.00 | 12.60 | 0 |
|  |  |  |  |  |  |
| Time [h] | OD420 | Glc [mM] | Ace [mM] | Lac [mM] | Pyr [mM] |
| 0.0 | 0.16 | n.d. | n.d. | n.d. | n.d. |
| 1.6 | 0.28 | n.d. | n.d. | n.d. | n.d. |
| 2.0 | 0.32 | n.d. | n.d. | n.d. | n.d. |
| 2.50 | 0.39 | n.d. | n.d. | n.d. | n.d. |
| 3.25 | 0.50 | n.d. | n.d. | n.d. | n.d. |
| 3.75 | 0.60 | n.d. | n.d. | n.d. | n.d. |
| 4.67 | 0.75 | n.d. | n.d. | n.d. | n.d. |
| 5.67 | 1.20 | n.d. | n.d. | n.d. | n.d. |
| 7.08 | 1.93 | n.d. | n.d. | n.d. | n.d. |
| 24.00 | 4.35 | n.d. | n.d. | n.d. | n.d. |

**Table 1: Average data for aerobic growth and by-product synthesis under aerobic conditions**

| **AEROBIC** | **MG1655**  **_(UQ,DMK,MK)_** | **AV34**  **_(UQ)_** | **AV33**  **_(DMK,MK)_** | **AV36**  **_(DMK)_** |
| --- | --- | --- | --- | --- |
| **Growth rate [h^-1^]** | 0.74 ± 0.07 | 0.75 ± 0.02 | 0.21 ± 0.02 | 0.38 ± 0.05 |
| **Organic acid** | **Yield [mol/mol_Glc_]** | | | |
| Acetate | 0.26 ± 0.08 | 0.29 ± 0.09 | 0.64 ± 0.12 | 0.61 ± 0.15 |
| Lactate | 0 | 0 | 1.45 ± 0.23 | 0,93 ± 0.25 |
| Pyruvate | 0 | 0 | 0.02 ± 0.00 | 0.03 ± 0.01 |

This table summarizes the data from the different growth curves displayed in sections 1-4. The table indicates specific growth rates during the exponential phase of growth given in doublings per hour. Product yields are given in mol product produced per mol of glucose consumed. Yields of 0 mean that the respective product could not be detected throughout the time course of the measurements.
