# Supplementary material for: All three quinone species play distinct roles in ensuring optimal growth under aerobic and fermentative conditions in E. coli K12
Source: PLoS One. 2018 Apr 3;13(4):e0194699. doi: 10.1371/journal.pone.0194699 (PMC5882134; doi:10.1371/journal.pone.0194699)
Supplement: S1 Dataset — This file contains the following: Suppl_data_aerobe.docx: Time course data for biomass and by-products from aerobic growth experiments. Suppl_data_anaerobe.docx: Time course data for biomass and by-products from anaerobic growth experiments. Suppl_data_CellRox: Individual data from oxidative stress measurements. Suppl_data_Quinone_aerobe.docx: Individual quinone concentrations from aerobic growth experiments. Suppl_data_Quinone_anaerobe.docx: Individual quinone concentrations from anaerobic growth experiments. (ZIP) [file pone.0194699.s002.zip › Suppl_Data/Suppl_data_anaerobe.docx]

1. Time course data of the different growth curves of MG1655 under anaerobic conditions.

| Time  [h] | | OD420 | | | | Glc [mM] | | | For [mM] | | | Pyr [mM] | | | Lac  [mM] | | | DHO  [mM] | | | Oro  [mM] | | | Ace  [mM] | | | | | | Fum  [mM] | | | Succ  [mM] | | | | | | EtOH  [mM] | | | |  |  |
| --- | --- | --- | --- | --- | --- | --- | --- | --- | --- | --- | --- | --- | --- | --- | --- | --- | --- | --- | --- | --- | --- | --- | --- | --- | --- | --- | --- | --- | --- | --- | --- | --- | --- | --- | --- | --- | --- | --- | --- | --- | --- | --- | --- | --- |
| *0.00* | | *0.19* | | | | *22.26* | | | *0.00* | | | *0.00* | | | *0.00* | | | *0.00* | | | *0.00* | | | *0.00* | | | | | | *0.00* | | | *0.00* | | | | | | *0.00* | | | |  |  |
| *1.50* | | *0.28* | | | | *21.37* | | | *0.50* | | | *0.00* | | | *0.00* | | | *0.00* | | | *0.01* | | | *0.44* | | | | | | *0.00* | | | *0.00* | | | | | | *0.33* | | | |  |  |
| *3.00* | | *0.55* | | | | *19.31* | | | *3.37* | | | *0.01* | | | *0.00* | | | *0.00* | | | *0.02* | | | *1.67* | | | | | | *0.01* | | | *0.45* | | | | | | *2.06* | | | |  |  |
| *4.75* | | *0.94* | | | | *14.72* | | | *10.81* | | | *0.07* | | | *0.00* | | | *0.00* | | | *0.06* | | | *4.76* | | | | | | *0.01* | | | *0.89* | | | | | | *6.05* | | | |  |  |
| *5.75* | | *1.62* | | | | *12.65* | | | *13.56* | | | *0.07* | | | *0.59* | | | *0.00* | | | *0.07* | | | *6.04* | | | | | | *0.01* | | | *1.16* | | | | | | *7.97* | | | |  |  |
| *7.00* | | *2.13* | | | | *6.23* | | | *18.29* | | | *0.19* | | | *2.15* | | | *0.01* | | | *0.09* | | | *9.08* | | | | | | *0.01* | | | *2.25* | | | | | | *11.39* | | | |  |  |
| *8.00* | | *2.52* | | | | *2.75* | | | *19.91* | | | *0.53* | | | *3.62* | | | *0.01* | | | *0.11* | | | *12.12* | | | | | | *0.02* | | | *2.96* | | | | | | *14.34* | | | |  |  |
| *24.00* | | *2.13* | | | | *0.13* | | | *17.91* | | | *0.10* | | | *5.82* | | | *0.01* | | | *0.11* | | | *14.17* | | | | | | *0.00* | | | *3.79* | | | | | | *16.38* | | | |  |  |
|  | |  | |  | |  | | |  | | |  | | |  | | |  | | |  | | |  | | | |  | | | | | | | |  | | | | | | | | |
| Time  [h] | | | OD420 | | | Glc [mM] | | | For [mM] | | | Pyr [mM] | | | Lac  [mM] | | | DHO  [mM] | | | Oro  [mM] | | | Ace  [mM] | | | | | | Fum  [mM] | | | Succ  [mM] | | | | | | EtOH  [mM] | | | | |  |
| *0.00* | | | *0.22* | | | *23.06* | | | *0.00* | | | *0.00* | | | *0.00* | | | *0.00* | | | *0.00* | | | *0.00* | | | | | | *0.00* | | | *0.00* | | | | | | *0.00* | | | | |  |
| *1.50* | | | *0.30* | | | *22.79* | | | *0.00* | | | *0.01* | | | *0.00* | | | *0.00* | | | *0.01* | | | *0.46* | | | | | | *0.00* | | | *0.00* | | | | | | *0.20* | | | | |  |
| *2.75* | | | *0.39* | | | *21.85* | | | *2.09* | | | *0.01* | | | *0.00* | | | *0.00* | | | *0.02* | | | *1.15* | | | | | | *0.00* | | | *0.20* | | | | | | *1.00* | | | | |  |
| *4.25* | | | *0.68* | | | *20.55* | | | *5.95* | | | *0.04* | | | *0.00* | | | *0.00* | | | *0.03* | | | *2.67* | | | | | | *0.00* | | | *0.61* | | | | | | *2.53* | | | | |  |
| *5.50* | | | *1.29* | | | *17.28* | | | *11.16* | | | *0.12* | | | *0.34* | | | *0.00* | | | *0.05* | | | *5.04* | | | | | | *0.00* | | | *0.85* | | | | | | *5.06* | | | | |  |
| *6.25* | | | *1.41* | | | *15.46* | | | *13.77* | | | *0.10* | | | *0.79* | | | *0.01* | | | *0.05* | | | *6.17* | | | | | | *0.00* | | | *0.70* | | | | | | *6.29* | | | | |  |
| *7.25* | | | *1.66* | | | *12.94* | | | *17.07* | | | *0.21* | | | *1.92* | | | *0.00* | | | *0.05* | | | *7.65* | | | | | | *0.00* | | | *0.84* | | | | | | *7.56* | | | | |  |
| *8.08* | | | *1.67* | | | *n.d.* | | | *n.d.* | | | *n.d.* | | | *n.d.* | | | *n.d.* | | | *n.d.* | | | *n.d.* | | | | | | *n.d.* | | | *n.d.* | | | | | | *n.d.* | | | | |  |
| *23* | | | *1.775* | | | *-0.03* | | | *27.49* | | | *0.20* | | | *7.27* | | | *0.01* | | | *0.07* | | | *14.51* | | | | | | *0.00* | | | *2.43* | | | | | | *14.88* | | | | |  |
|  | |  | |  | |  | | |  | | |  | | |  | | |  | | |  | | |  | | | |  | | | | | | | | |  | | | |  |  |  |  |
| Time  [h] | | | | OD420 | | | Glc [mM] | | | For [mM] | | | Pyr [mM] | | | Lac  [mM] | | | DHO  [mM] | | | Oro  [mM] | | | | Ace  [mM] | | | | Fum  [mM] | | | | Succ  [mM] | | | | | EtOH  [mM] | | | | |  |
| *0.00* | | | | *0.46* | | | *15.45* | | | *n.d* | | | *0.00* | | | *0.00* | | | *n.d.* | | | *0.00* | | | | *0.00* | | | | *0.00* | | | | *n.d.* | | | | | *0.00* | | | | |  |
| *2.25* | | | | *1.13* | | | *11.91* | | | *n.d.* | | | *0.03* | | | *0.00* | | | *n.d.* | | | *0.05* | | | | *2.53* | | | | *0.01* | | | | *n.d.* | | | | | *0.75* | | | | |  |
| *4.25* | | | | *2.01* | | | *6.87* | | | *n.d.* | | | *0.13* | | | *0.45* | | | *n.d.* | | | *0.10* | | | | *7.83* | | | | *0.03* | | | | *n.d.* | | | | | *7.45* | | | | |  |
| *24.00* | | | | *2.85* | | | *0.17* | | | *n.d.* | | | *0.00* | | | *4.80* | | | *n.d.* | | | *0.13* | | | | *12.67* | | | | *0.00* | | | | *n.d.* | | | | | *10.10* | | | | |  |
|  | |  | |  | |  | | |  | | |  | | |  | | |  | | |  | | |  | | | |  | | | | | | | | |  | | | |  |  |  |  |
| Time  [h] | | | OD420 | | | | Glc [mM] | | | For [mM] | | | Pyr [mM] | | | Lac  [mM] | | | DHO  [mM] | | | Oro  [mM] | | | Ace  [mM] | | | | Fum  [mM] | | | Succ  [mM] | | | | | | EtOH  [mM] | | | |  |  |  |
| *0.00* | | | *0.11* | | | | *22.03* | | | *0.00* | | | *0.00* | | | *n.d.* | | | *n.d.* | | | *n.d.* | | | *0.00* | | | | *0.00* | | | *0.00* | | | | | | *0.00* | | | |  |  |  |
| *1.08* | | | *0.24* | | | | *19.65* | | | *0.00* | | | *0.00* | | | *n.d.* | | | *n.d.* | | | *n.d.* | | | *0.00* | | | | *0.01* | | | *0.15* | | | | | | *0.21* | | | |  |  |  |
| *2.17* | | | *0.37* | | | | *19.86* | | | *2.54* | | | *0.02* | | | *n.d.* | | | *n.d.* | | | *n.d.* | | | *2.27* | | | | *0.00* | | | *0.22* | | | | | | *0.57* | | | |  |  |  |
| *3.67* | | | *0.67* | | | | *15.94* | | | *3.63* | | | *0.01* | | | *n.d.* | | | *n.d.* | | | *n.d.* | | | *2.36* | | | | *0.01* | | | *0.26* | | | | | | *1.42* | | | |  |  |  |
| *5.08* | | | *1.24* | | | | *13.05* | | | *9.21* | | | *0.09* | | | *n.d.* | | | *n.d.* | | | *n.d.* | | | *5.65* | | | | *0.02* | | | *0.77* | | | | | | *1.99* | | | |  |  |  |
| *6.12* | | | *1.82* | | | | *8.58* | | | *16.40* | | | *0.10* | | | *n.d.* | | | *n.d.* | | | *n.d.* | | | *10.12* | | | | *0.03* | | | *1.45* | | | | | | *6.23* | | | |  |  |  |
| *7.00* | | | *2.32* | | | | *n.d.* | | | *n.d.* | | | *n.d.* | | | *n.d.* | | | *n.d.* | | | *n.d.* | | | *n.d.* | | | | *n.d.* | | | *n.d.* | | | | | | *n.d.* | | | |  |  |  |
| *23.25* | | | *2.47* | | | | *0.02* | | | *4.10* | | | *0.02* | | | *n.d.* | | | *n.d.* | | | *n.d.* | | | *19.89* | | | | *0.01* | | | *2.34* | | | | | | *8.04* | | | |  |  |  |
|  | | | |  | | |  | | |  | | |  | |  | | |  | | |  | | |  | | | |  | | |  | | | | | |  | | | |  |  |  |  |
|  | | | |  | | |  | | |  | | |  | |  | | |  | | |  | | |  | | | |  | | |  | | | | | |  | | | |  |  |  |  |
| Time  [h] | | | | OD420 | | | Glc [mM] | | | For [mM] | | | Pyr [mM] | | Lac  [mM] | | | DHO  [mM] | | | Oro  [mM] | | | Ace  [mM] | | | | Fum  [mM] | | | Succ  [mM] | | | | | | EtOH  mM] | | | |  |  |  |  |
| *0.00* | | | | *0.21* | | | *23.70* | | | *0.00* | | | *0.00* | | *n.d.* | | | *n.d.* | | | *0.00* | | | *0.00* | | | | *0.00* | | | *0.00* | | | | | | *n.d.* | | | |  |  |  |  |
| *1.00* | | | | *0.26* | | | *17.49* | | | *0.00* | | | *0.00* | | *n.d.* | | | *n.d.* | | | *0.00* | | | *0.23* | | | | *0.00* | | | *0.00* | | | | | | *n.d.* | | | |  |  |  |  |
| *2.00* | | | | *0.38* | | | *17.06* | | | *0.48* | | | *0.00* | | *n.d.* | | | *n.d.* | | | *0.01* | | | *0.70* | | | | *0.00* | | | *0.00* | | | | | | *n.d.* | | | |  |  |  |  |
| *3.00* | | | | *0.55* | | | *17.07* | | | *2.00* | | | *0.01* | | *n.d.* | | | *n.d.* | | | *0.02* | | | *1.48* | | | | *0.00* | | | *0.10* | | | | | | *n.d.* | | | |  |  |  |  |
| *4.00* | | | | *0.79* | | | *15.07* | | | *4.10* | | | *0.03* | | *n.d.* | | | *n.d.* | | | *0.04* | | | *2.96* | | | | *0.01* | | | *0.31* | | | | | | *n.d.* | | | |  |  |  |  |
| *5.00* | | | | *1.41* | | | *13.27* | | | *7.69* | | | *0.05* | | *n.d.* | | | *n.d.* | | | *0.06* | | | *5.40* | | | | *0.02* | | | *1.13* | | | | | | *n.d.* | | | |  |  |  |  |
| *6.00* | | | | *1.84* | | | *9.54* | | | *11.58* | | | *0.09* | | *n.d.* | | | *n.d.* | | | *0.08* | | | *8.97* | | | | *0.03* | | | *1.43* | | | | | | *n.d.* | | | |  |  |  |  |
| *7.00* | | | | *2.61* | | | *3.64* | | | *15.80* | | | *0.20* | | *n.d.* | | | *n.d.* | | | *0.12* | | | *12.78* | | | | *0.03* | | | *2.54* | | | | | | *n.d.* | | | |  |  |  |  |
| *7.92* | | | | *3.16* | | | *0.00* | | | *16.58* | | | *0.21* | | *n.d.* | | | *n.d.* | | | *0.13* | | | *13.57* | | | | *0.03* | | | *3.44* | | | | | | *n.d.* | | | |  |  |  |  |
| *8.42* | | | | *2.69* | | | *0.00* | | | *15.70* | | | *0.02* | | *n.d.* | | | *n.d.* | | | *0.13* | | | *15.06* | | | | *0.02* | | | *3.55* | | | | | | *n.d.* | | | |  |  |  |  |
| *23.83* | | | | *2.36* | | | *0.00* | | | *1.61* | | | *0.03* | | *n.d.* | | | *n.d.* | | | *0.14* | | | *18.30* | | | | *0.00* | | | *3.94* | | | | | | *n.d.* | | | |  |  |  |  |

2. Time course data of the different growth curves of AV34 under anaerobic conditions.

| Time  [h] | OD420 | Glc [mM] | For [mM] | Pyr [mM] | Lac  [mM] | DHO  [mM] | Oro  [mM] | Ace  [mM] | Fum  [mM] | Succ  [mM] | EtOH  [mM] |
| --- | --- | --- | --- | --- | --- | --- | --- | --- | --- | --- | --- |
| 0.00 | 0.22 | 21.76 | n.d. | n.d. | n.d. | n.d. | n.d. | n.d. | n.d. | n.d. | n.d. |
| 1.50 | 0.28 | 21.65 | n.d. | n.d. | n.d. | n.d. | n.d. | n.d. | n.d. | n.d. | n.d. |
| 2.75 | 0.31 | 20.98 | n.d. | n.d. | n.d. | n.d. | n.d. | n.d. | n.d. | n.d. | n.d. |
| 4.25 | 0.33 | 20.42 | n.d. | n.d. | n.d. | n.d. | n.d. | n.d. | n.d. | n.d. | n.d. |
| 5.50 | 0.32 | 19.63 | n.d. | n.d. | n.d. | n.d. | n.d. | n.d. | n.d. | n.d. | n.d. |
| 7.00 | 0.33 | 19.97 | n.d. | n.d. | n.d. | n.d. | n.d. | n.d. | n.d. | n.d. | n.d. |
| 23.50 | 0.66 | 12.60 | n.d. | n.d. | n.d. | n.d. | n.d. | n.d. | n.d. | n.d. | n.d. |
|  |  |  |  |  |  |  |  |  |  |  |  |
| Time  [h] | OD420 | Glc [mM] | For [mM] | Pyr [mM] | Lac  [mM] | DHO  [mM] | Oro  [mM] | Ace  [mM] | Fum  [mM] | Succ  [mM] | EtOH  [mM] |
| 0.00 | 0.23 | 19.52 | n.d. | n.d. | n.d. | n.d. | n.d. | n.d. | n.d. | n.d. | n.d. |
| 1.50 | 0.26 | 19.86 | n.d. | n.d. | n.d. | n.d. | n.d. | n.d. | n.d. | n.d. | n.d. |
| 2.75 | 0.31 | 20.42 | n.d. | n.d. | n.d. | n.d. | n.d. | n.d. | n.d. | n.d. | n.d. |
| 4.50 | 0.30 | 18.40 | n.d. | n.d. | n.d. | n.d. | n.d. | n.d. | n.d. | n.d. | n.d. |
| 5.25 | 0.31 | 18.40 | 1.49 | 0.00 | n.i.c. | 0.04 | 0.00 | 0.82 | -0.03 | 0.00 | n.i.c. |
| 6.25 | 0.31 | 17.61 | 2.28 | 0.00 | n.i.c. | 0.04 | 0.00 | 1.03 | 1.78 | 0.00 | n.i.c. |
| 23.75 | 0.92 | 4.21 | 16.16 | 0.16 | n.i.c. | 0.36 | 0.00 | 7.72 | 2.66 | 0.00 | n.i.c. |
| 29.75 | 1.21 | 1.49 | 14.30 | 0.40 | n.i.c. | 0.61 | 0.00 | 9.86 | 1.78 | 0.00 | n.i.c. |
| 48.00 | 1.40 | -0.17 | 10.13 | 0.24 | n.i.c. | 0.67 | 0.00 | 10.64 | 2.32 | 0.00 | n.i.c. |
|  |  |  |  |  |  |  |  |  |  |  |  |
| Time  [h] | OD420 | Glc [mM] | For [mM] | Pyr [mM] | Lac  [mM] | DHO  [mM] | Oro  [mM] | Ace  [mM] | Fum  [mM] | Succ  [mM] | EtOH  [mM] |
| 0.00 | 0.24 | 20.86 | n.i.c. | n.i.c. | n.i.c. | n.i.c. | n.i.c. | n.i.c. | n.i.c. | n.i.c. | 0.00 |
| 8.00 | 0.29 | 18.85 | n.i.c. | n.i.c. | n.i.c. | n.i.c. | n.i.c. | n.i.c. | n.i.c. | n.i.c. | 1.78 |
| 9.50 | 0.29 | 18.74 | n.d. | n.d. | n.d. | n.d. | n.d. | n.d. | n.d. | n.d. | 2.66 |
| 9.75 | 0.30 | 18.00 | n.d. | n.d. | n.d. | n.d. | n.d. | n.d. | n.d. | n.d. | 1.78 |
| 11.50 | 0.35 | 18.00 | n.d. | n.d. | n.d. | n.d. | n.d. | n.d. | n.d. | n.d. | 2.32 |
| 12.50 | 0.34 | 18.32 | n.d. | n.d. | n.d. | n.d. | n.d. | n.d. | n.d. | n.d. | 2.73 |
| 14.00 | 0.38 | 16.30 | n.d. | n.d. | n.d. | n.d. | n.d. | n.d. | n.d. | n.d. | 1.38 |
| 15.00 | 0.38 | 16.83 | n.d. | n.d. | n.d. | n.d. | n.d. | n.d. | n.d. | n.d. | 4.48 |
| 16.00 | 0.43 | 15.77 | n.i.c. | n.i.c. | n.i.c. | n.i.c. | n.i.c. | n.i.c. | n.i.c. | n.i.c. | 3.60 |
| 32.50 | 0.81 | 4.51 | n.i.c. | n.i.c. | n.i.c. | n.i.c. | n.i.c. | n.i.c. | n.i.c. | n.i.c. | 11.21 |
| 37.00 | 0.79 | 3.56 | n.d. | n.d. | n.d. | n.d. | n.d. | n.d. | n.d. | n.d. | n.d. |
|  |  |  |  |  |  |  |  |  |  |  |  |
| Time  [h] | OD420 | Glc [mM] | For [mM] | Pyr [mM] | Lac  [mM] | DHO  [mM] | Oro  [mM] | Ace  [mM] | Fum  [mM] | Succ  [mM] | EtOH  [mM] |
| 0.00 | 0.638 | 22.35 | 0.00 | 0.00 | 0.00 | 0.00 | 0.00 | 0.00 | 0.00 | 0.00 | 0.00 |
| 13.00 | 0.876 | 12.79 | 14.76 | 0.00 | 0.28 | 0.28 | 0.00 | 6.21 | 0.07 | 0.00 | 7.37 |
| 14.00 | 1.22 | 13.01 | 16.09 | 0.00 | 0.31 | 0.32 | 0.00 | 6.76 | 0.08 | 0.00 | 9.60 |
| 15.00 | 1.345 | 11.63 | 17.29 | 0.06 | 0.57 | 0.33 | 0.00 | 7.55 | 0.09 | 0.00 | 10.27 |
| 16.00 | 1.495 | 10.78 | 17.77 | 0.11 | 1.19 | 0.36 | 0.00 | 8.15 | 0.10 | 0.00 | 9.87 |
| 17.75 | 1.515 | 9.08 | 19.20 | 0.17 | 1.52 | 0.43 | 0.00 | 8.91 | 0.11 | 0.00 | 9.33 |
| 19.75 | 1.605 | 7.06 | 19.80 | 0.29 | 2.55 | 0.51 | 0.00 | 9.63 | 0.12 | 0.00 | 12.56 |
| 37.25 | 1.485 | -0.05 | 19.75 | 0.29 | 2.57 | 0.51 | 0.00 | 9.67 | 0.12 | 0.00 | 13.70 |
|  |  |  |  |  |  |  |  |  |  |  |  |
| Time  [h] | OD420 | Glc [mM] | For [mM] | Pyr [mM] | Lac  [mM] | DHO  [mM] | Oro  [mM] | Ace  [mM] | Fum  [mM] | Succ  [mM] | EtOH  [mM] |
| 0.00 | 0.58 | 45.44 | 0.00 | 0.00 | 0.00 | 0.00 | 0.00 | 0.00 | 0.00 | 0.00 | 0.00 |
| 12.00 | 1.29 | 34.40 | 17.94 | 0.04 | 0.82 | 0.34 | 0.00 | 8.41 | 0.10 | 0.00 | 7.21 |
| 13.33 | 1.35 | 32.95 | 19.77 | 0.05 | 1.15 | 0.39 | 0.00 | 9.62 | 0.11 | 0.00 | 9.29 |
| 15.00 | 1.37 | 31.28 | 20.70 | 0.07 | 1.46 | 0.47 | 0.00 | 10.67 | 0.12 | 0.00 | 9.81 |
| 17.50 | 1.39 | 28.82 | 21.41 | 0.11 | 2.47 | 0.49 | 0.00 | 11.88 | 0.14 | 0.00 | 10.46 |
| 18.33 | 1.44 | 29.27 | 22.70 | 0.18 | 3.15 | 0.55 | 0.00 | 12.28 | 0.14 | 0.00 | 11.57 |
| 19.33 | 1.49 | 26.82 | 21.30 | 0.20 | 3.72 | 0.50 | 0.00 | 12.86 | 0.14 | 0.00 | 11.82 |
| 41.00 | 0.96 | 18.79 | 21.95 | 0.48 | 14.99 | 0.75 | 0.00 | 15.43 | 0.12 | 0.00 | 14.81 |
|  |  |  |  |  |  |  |  |  |  |  |  |
| Time  [h] | OD420 | Glc [mM] | For [mM] | Pyr [mM] | Lac  [mM] | DHO  [mM] | Oro  [mM] | Ace  [mM] | Fum  [mM] | Succ  [mM] | EtOH  [mM] |
| 0.00 | 0.63 | 44.88 | n.d. | 0.00 | 0.00 | 0.00 | 0.00 | 0.00 | 0.00 | 0.00 | 0.00 |
| 12.00 | 1.27 | 33.84 | n.d. | 0.04 | 1.24 | 0.33 | 0.00 | 9.45 | 0.11 | 0.00 | 7.99 |
| 13.33 | 1.33 | 31.61 | n.d. | 0.05 | 1.46 | 0.37 | 0.00 | 10.17 | 0.13 | 0.00 | 8.64 |
| 15.00 | 1.41 | 28.15 | n.d. | 0.13 | 3.51 | 0.43 | 0.00 | 11.85 | 0.16 | 0.00 | 10.33 |
| 17.50 | 1.43 | 27.48 | n.d. | 0.19 | 4.21 | 0.52 | 0.00 | 12.33 | 0.15 | 0.00 | 11.05 |
| 18.33 | 1.56 | 26.70 | n.d. | 0.13 | 3.34 | 0.53 | 0.00 | 12.63 | 0.16 | 0.00 | 11.95 |
| 19.33 | 1.42 | 26.59 | n.d. | 0.23 | 5.27 | 0.58 | 0.00 | 12.34 | 0.14 | 0.00 | 11.31 |
| 41.00 | 1.19 | 17.00 | n.d. | 0.45 | 15.99 | 0.72 | 0.00 | 15.75 | 0.14 | 0.00 | 14.55 |
|  |  |  |  |  |  |  |  |  |  |  |  |
| Time  [h] | OD420 | Glc [mM] | For [mM] | Pyr [mM] | Lac  [mM] | DHO  [mM] | Oro  [mM] | Ace  [mM] | Fum  [mM] | Succ  [mM] | EtOH  [mM] |
| 0.00 | 0.267 | 44.54 | 0.00 | 0.00 | n.d. | 0.00 | 0.00 | 0.00 | 0.00 | 0.00 | 0.00 |
| 12.67 | 0.397 | 42.52 | 4.89 | 0.00 | n.d. | 0.01 | 0.00 | 2.06 | 0.01 | 0.00 | 2.79 |
| 16.00 | 0.401 | 39.34 | 9.34 | 0.00 | n.d. | 0.06 | 0.00 | 4.22 | 0.03 | 0.00 | 3.87 |
| 17.00 | 0.674 | 37.85 | 10.95 | 0.00 | n.d. | 0.06 | 0.00 | 5.47 | 0.03 | 0.00 | 5.62 |
| 18.00 | 0.82 | 37.53 | 9.80 | 0.00 | n.d. | 0.05 | 0.00 | 4.08 | 0.02 | 0.00 | 6.63 |
| 18.92 | 0.8675 | 36.90 | 12.49 | 0.00 | n.d. | 0.08 | 0.00 | 5.99 | 0.04 | 0.00 | 5.35 |
| 19.08 | 0.8875 | 37.00 | 12.40 | 0.00 | n.d. | 0.07 | 0.00 | 6.43 | 0.05 | 0.00 | 8.52 |
| 19.25 | 0.8725 | 37.11 | 13.08 | 0.00 | n.d. | 0.09 | 0.00 | 6.24 | 0.05 | 0.00 | 5.83 |
| 19.92 | 0.9575 | 37.21 | 13.05 | 0.00 | n.d. | 0.27 | 0.00 | 6.27 | 0.05 | 0.00 | 8.38 |
| 19.92 | 0.925 | 34.88 | 14.04 | 0.00 | n.d. | 0.27 | 0.00 | 5.77 | 0.05 | 0.00 | 9.13 |
| 19.92 | 0.965 | 34.67 | 20.14 | 0.00 | n.d. | 0.27 | 0.00 | 6.27 | 0.11 | 0.00 | 8.86 |
| 35.92 | 1.235 | 25.32 | 19.16 | 0.43 | n.d. | 0.30 | 0.00 | 10.60 | 0.11 | 0.00 | 15.86 |
| 35.92 | 1.26 | 25.00 | 19.50 | 0.48 | n.d. | 0.30 | 0.00 | 10.97 | 0.11 | 0.00 | 15.86 |
| 35.92 | 1.29 | 23.20 | 0.00 | 0.00 | n.d. | 0.30 | 0.00 | 11.30 | 0.09 | 0.00 | 15.19 |

3. Time course data of the different growth curves of AV33 under anaerobic conditions.

| Time  [h] | OD420 | Glc [mM] | For [mM] | Pyr [mM] | Lac  [mM] | DHO [mM] | Oro  [mM] | Ace  [mM] | Fum  [mM] | Succ  [mM] | EtOH [mM] |
| --- | --- | --- | --- | --- | --- | --- | --- | --- | --- | --- | --- |
| 0.00 | 0.20 | 20.33 | 0.00 | 0.00 | 0.00 | 0.000 | 0.00 | 0.00 | 0.00 | 0.00 | 0.00 |
| 1.50 | 0.27 | 19.26 | 0.00 | 0.02 | 0.61 | 0.000 | 0.00 | 0.00 | 0.00 | 0.00 | 0.06 |
| 3.00 | 0.42 | 18.12 | 0.99 | 0.01 | 0.99 | 0.002 | 0.01 | 0.57 | 0.00 | 0.00 | 0.84 |
| 4.58 | 0.80 | 16.09 | 5.00 | 0.01 | 0.97 | 0.002 | 0.03 | 2.24 | 0.01 | 0.41 | 3.34 |
| 5.33 | 1.08 | 15.09 | 5.78 | 0.01 | 0.97 | 0.002 | 0.04 | 2.71 | 0.01 | 0.41 | 4.35 |
| 6.33 | 1.33 | 11.94 | 9.58 | 0.07 | 1.03 | 0.005 | 0.05 | 3.99 | 0.01 | 0.90 | 6.25 |
| 7.33 | 2.00 | 8.68 | 13.06 | 0.13 | 1.28 | 0.006 | 0.07 | 6.28 | 0.03 | 1.35 | 9.99 |
| 8.58 | 2.01 | 5.32 | 14.91 | 0.29 | 2.27 | 0.008 | 0.07 | 7.80 | 0.03 | 2.05 | 12.38 |
| 22.58 | 1.58 | 0.17 | 14.35 | 0.16 | 5.07 | 0.010 | 0.08 | 11.19 | 0.02 | 0.47 | 14.96 |
|  |  |  |  |  |  |  |  |  |  |  |  |
| Time [h] | OD420 | Glc [mM] | For [mM] | Pyr [mM] | Lac  [mM] | DHO  [mM] | Oro  [mM] | Ace  [mM] | Fum  [mM] | Succ  [mM] | EtOH  [mM] |
| 0.00 | 0.13 | 20.50 | 0.00 | 0.00 | 0.00 | .000 | 0.00 | 0.00 | 0.00 | 0.00 | 0.00 |
| 1.75 | 0.30 | 20.35 | 0.00 | 0.01 | 0.93 | .000 | 0.00 | 0.00 | 0.00 | 0.00 | 0.11 |
| 3.00 | 0.41 | 19.37 | 1.61 | 0.00 | 1.09 | .000 | 0.01 | 0.77 | 0.00 | 0.00 | 0.86 |
| 4.50 | 0.67 | 17.26 | 5.23 | 0.02 | 1.15 | .003 | 0.02 | 2.25 | 0.01 | 0.24 | 2.54 |
| 5.50 | 1.04 | 14.80 | 9.85 | 0.06 | 1.29 | .005 | 0.03 | 4.41 | 0.02 | 0.74 | 4.77 |
| 6.50 | 1.25 | 13.50 | 12.58 | 0.05 | 1.25 | .006 | 0.04 | 5.64 | 0.03 | 0.77 | 6.94 |
| 7.50 | 1.41 | 10.38 | 15.15 | 0.09 | 1.44 | .008 | 0.05 | 6.99 | 0.04 | 1.34 | 8.31 |
| 8.58 | 1.83 | 6.94 | 18.96 | 0.22 | 1.95 | .009 | 0.06 | 9.42 | 0.07 | 2.07 | 12.04 |
| 23.25 | 1.81 | 0.21 | 9.17 | 0.20 | 5.17 | .009 | 0.07 | 12.99 | 0.00 | 4.30 | 15.32 |
|  |  |  |  |  |  |  |  |  |  |  |  |
| Time [h] | OD420 | Glc [mM] | For [mM] | Pyr [mM] | Lac  [mM] | DHO  [mM} | Oro  [mM] | Ace  [mM] | Fum  [mM] | Succ  [mM] | EtOH  [mM] |
| 0.00 | 0.17 | 23.37 | 0.00 | 0.00 | 0.00 | n.d. | 0.00 | 0.00 | 0.00 | 0.00 | n.i.c |
| 0.92 | 0.19 | 22.31 | 0.00 | 0.00 | 0.00 | n.d. | 0.00 | 0.00 | 0.00 | 0.00 | n.i.c |
| 1.83 | 0.26 | 22.84 | 0.82 | 0.00 | 0.00 | n.d. | 0.00 | 0.00 | 0.00 | 0.00 | n.i.c |
| 3.25 | 0.42 | 18.10 | 2.92 | 0.00 | 0.00 | n.d. | 0.01 | 1.37 | 0.00 | 0.00 | n.i.c |
| 4.75 | 0.74 | 13.85 | 6.99 | 0.04 | 0.42 | n.d. | 0.02 | 3.70 | 0.01 | 0.00 | n.i.c |
| 6.25 | 1.36 | 10.64 | 14.28 | 0.13 | 0.58 | n.d. | 0.03 | 6.94 | 0.02 | 0.97 | n.i.c |
| 7.25 | 1.74 | 8.11 | 19.10 | 0.17 | 0.99 | n.d. | 0.04 | 9.33 | 0.04 | 1.48 | n.i.c |
| 25.25 | 2.17 | 0.18 | 22.41 | 0.00 | 4.80 | n.d. | 0.06 | 15.10 | 0.00 | 2.81 | n.i.c |
|  |  |  |  |  |  |  |  |  |  |  |  |
| Time [h] | OD420 | Glc [mM] | For [mM] | Pyr [mM] | Lac  [mM] | DHO  [mM] | Oro  [mM] | Ace  [mM] | Fum  [mM] | Succ  [mM] | EtOH  [mM] |
| 0.00 | 0.17 | 19.78 | 0.00 | 0.00 | 0.00 | n.d. | 0.00 | 0.00 | 0.00 | 0.00 | 0.00 |
| 1.00 | 0.20 | 20.52 | 0.00 | 0.00 | 0.54 | n.d. | 0.00 | 0.00 | 0.00 | 0.00 | 0.27 |
| 2.00 | 0.25 | 18.46 | 0.00 | 0.00 | 0.97 | n.d. | 0.00 | 0.00 | 0.00 | 0.00 | 0.37 |
| 3.00 | 0.31 | n.d. | 0.00 | 0.01 | 1.52 | n.d. | 0.00 | 0.23 | 0.00 | 0.00 | 0.33 |
| 4.00 | 0.39 | n.d. | 0.00 | 0.01 | 2.87 | n.d. | 0.00 | 0.32 | 0.00 | 0.00 | 0.14 |
| 5.00 | 0.52 | 15.97 | 0.17 | 0.01 | 4.26 | n.d. | 0.00 | 0.54 | 0.00 | 0.00 | 0.94 |
| 6.00 | 0.63 | n.d. | 0.90 | 0.01 | 4.71 | n.d. | 0.01 | 1.26 | 0.00 | 0.00 | 1.90 |
| 7.00 | 0.88 | 14.97 | 2.93 | 0.02 | 5.70 | n.d. | 0.02 | 2.62 | 0.00 | 0.00 | 2.38 |
| 7.75 | 1.10 | 14.02 | 5.59 | 0.05 | 4.96 | n.d. | 0.03 | 4.24 | 0.01 | 0.33 | 7.09 |
| 8.50 | 1.47 | 9.85 | 8.50 | 0.08 | 5.13 | n.d. | 0.04 | 5.99 | 0.02 | 0.59 | 6.97 |
| 9.25 | 1.74 | 8.10 | 11.07 | 0.10 | 5.59 | n.d. | 0.05 | 7.58 | 0.04 | 0.85 | 11.35 |
| 23.25 | 2.10 | 0.00 | 11.14 | 0.03 | 8.97 | n.d. | 0.07 | 14.72 | 0.01 | 1.75 | 13.40 |

4. Time course data of the different growth curves of AV36 under anaerobic conditions.

| Time [h] | OD420 | Glc [mM] | For [mM] | Pyr [mM] | Lac  [mM] | DHO  [mM] | Oro  [mM] | Ace  [mM] | Fum  [mM] | Succ  [mM] | EtOH [mM] |
| --- | --- | --- | --- | --- | --- | --- | --- | --- | --- | --- | --- |
| 0.00 | 0.22 | 21.97 | 0.00 | 0.00 | 0.00 | 0.000 | 0.00 | 0.00 | 0.00 | 0.00 | 0.00 |
| 1.50 | 0.27 | 21.04 | 0.00 | 0.00 | 0.34 | 0.000 | 0.00 | 0.00 | 0.00 | 0.00 | 0.15 |
| 3.00 | 0.42 | 19.74 | 1.37 | 0.00 | 0.47 | 0.000 | 0.01 | 0.83 | 0.00 | 0.10 | 0.76 |
| 4.00 | 0.68 | 17.64 | 3.65 | 0.02 | 0.41 | 0.002 | 0.02 | 1.80 | 0.00 | 0.30 | 1.76 |
| 5.00 | 0.95 | 15.68 | 6.82 | 0.02 | 0.41 | 0.003 | 0.04 | 3.11 | 0.01 | 0.53 | 3.07 |
| 6.00 | 1.09 | 12.90 | 10.07 | 0.04 | 0.48 | 0.006 | 0.05 | 4.92 | 0.01 | 0.97 | 4.41 |
| 7.00 | 1.19 | 7.81 | 13.44 | 0.08 | 0.86 | 0.007 | 0.07 | 6.86 | 0.02 | 1.39 | 5.89 |
| 8.00 | 1.23 | 5.96 | 15.46 | 0.20 | 1.70 | 0.007 | 0.07 | 8.39 | 0.04 | 2.02 | 7.44 |
| 24.00 | 1.02 | 0.44 | 15.77 | 0.11 | 4.52 | 0.017 | 0.09 | 11.41 | 0.00 | 2.87 | 9.93 |
|  |  |  |  |  |  |  |  |  |  |  |  |
| Time [h] | OD420 | Glc [mM] | For [mM] | Pyr [mM] | Lac  [mM] | DHO [mM] | Oro  [mM] | Ace  [mM] | Fum  [mM] | Succ  [mM] | EtOH [mM] |
| 0.00 | 0.31 | 20.15 | 0.00 | 0.00 | 0.00 | 0.000 | 0.00 | 0.00 | 0.00 | 0.00 | 0.00 |
| 1.00 | 0.40 | 18.30 | 0.00 | 0.00 | 0.35 | 0.000 | 0.00 | 0.00 | 0.00 | 0.00 | 0.37 |
| 2.00 | 0.51 | 17.98 | 1.67 | 0.00 | 0.32 | 0.000 | 0.01 | 1.31 | 0.00 | 0.00 | 0.64 |
| 3.00 | 0.67 | 17.44 | 2.37 | 0.02 | 0.40 | 0.003 | 0.01 | 1.34 | 0.00 | 0.30 | 1.78 |
| 4.00 | 1.03 | 14.56 | 6.02 | 0.03 | 0.43 | 0.006 | 0.03 | 3.01 | 0.01 | 0.47 | 3.13 |
| 5.00 | 1.30 | 12.24 | 8.82 | 0.04 | 0.39 | 0.008 | 0.04 | 4.30 | 0.01 | 0.76 | 5.08 |
| 5.67 | 1.49 | 10.34 | 9.21 | 0.06 | 0.46 | 0.007 | 0.05 | 4.73 | 0.02 | 0.98 | 5.35 |
| 6.50 | 1.79 | 7.96 | 11.13 | 0.08 | 0.85 | 0.010 | 0.07 | 5.56 | 0.04 | 1.57 | 5.75 |
| 23.50 | 1.58 | 0.00 | 12.75 | 0.11 | 5.08 | 0.020 | 0.09 | 11.35 | 0.00 | 2.97 | 7.40 |
|  |  |  |  |  |  |  |  |  |  |  |  |
| Time [h] | OD420 | Glc [mM] | For [mM] | Pyr [mM] | Lac  [mM] | DHO [mM] | Oro  [mM] | Ace  [mM] | Fum  [mM] | Succ  [mM] | EtOH [mM] |
| 0.00 | 0.17 | 22.57 | 0.00 | 0.00 | 0.00 | n.d. | 0.00 | 0.00 | 0.00 | 0.00 | 0.00 |
| 0.92 | 0.23 | 23.63 | 0.00 | 0.00 | 0.00 | n.d. | 0.00 | 0.00 | 0.00 | 0.00 | 0.47 |
| 1.83 | 0.33 | 22.04 | 0.77 | 0.00 | 0.00 | n.d. | 0.00 | 0.00 | 0.00 | 0.00 | 1.15 |
| 3.25 | 0.47 | 18.10 | 3.14 | 0.00 | 0.00 | n.d. | 0.01 | 1.92 | 0.00 | 0.00 | 2.16 |
| 4.75 | 0.90 | 14.12 | 8.20 | 0.04 | 0.00 | n.d. | 0.03 | 4.60 | 0.01 | 0.00 | 3.54 |
| 6.25 | 1.75 | 8.67 | 16.72 | 0.16 | 1.17 | n.d. | 0.07 | 8.63 | 0.02 | 1.09 | 6.01 |
| 7.25 | 2.06 | 4.73 | 20.22 | 0.29 | 3.30 | n.d. | 0.08 | 10.48 | 0.03 | 1.53 | 7.45 |
| 25.25 | 2.30 | 0.23 | 15.67 | 0 | 5.67 | n.d. | 0.06 | 17.28 | 0.00 | 2.42 | 0.14 |
|  |  |  |  |  |  |  |  |  |  |  |  |
| Time [h] | OD420 | Glc [mM] | For [mM] | Pyr [mM] | Lac  [mM] | DHO [mM] | Oro  [mM] | Ace  [mM] | Fum  [mM] | Succ  [mM] | EtOH [mM] |
| 0.00 | 0.19 | 18.69 | 0.00 | 0.00 | 0.00 | n.d. | 0.00 | 0.00 | 0.00 | 0.00 | 0.00 |
| 1.00 | 0.23 | 17.90 | 0.00 | 0.01 | 0.00 | n.d. | 0.00 | 0.02 | 0.00 | 0.00 | 0.20 |
| 2.00 | 0.30 | 17.79 | 1.56 | 0.05 | 0.00 | n.d. | 0.00 | 0.38 | 0.00 | 0.00 | 0.26 |
| 3.00 | 0.42 | 18.48 | 2.09 | 0.04 | 0.02 | n.d. | 0.01 | 1.01 | 0.00 | 0.00 | 0.59 |
| 4.00 | 0.62 | 17.05 | 5.03 | 0.09 | 0.45 | n.d. | 0.02 | 2.06 | 0.00 | 0.00 | 1.96 |
| 5.00 | 0.96 | 15.54 | 8.37 | 0.21 | 0.54 | n.d. | 0.03 | 3.94 | 0.00 | 0.27 | 3.36 |
| 6.00 | 1.51 | 11.99 | 11.16 | 0.31 | 0.54 | n.d. | 0.05 | 6.55 | 0.00 | 0.66 | 4.76 |
| 7.00 | 2.02 | 7.78 | 11.13 | 0.46 | 1.08 | n.d. | 0.07 | 9.36 | 0.00 | 1.38 | 9.11 |
| 7.75 | 2.70 | 4.42 | 10.60 | 0.62 | 2.31 | n.d. | 0.08 | 11.83 | 0.00 | 1.86 | 8.48 |
| 8.50 | 2.78 | 1.22 | 10.47 | 0.59 | 4.40 | n.d. | 0.09 | 13.74 | 0.00 | 2.49 | 11.74 |
| 9.25 | 2.85 | 0.00 | 10.26 | 0.58 | 5.35 | n.d. | 0.09 | 14.72 | 0.00 | 2.86 | 12.60 |
| 23.25 | 2.69 | 0.00 | 6.72 | 0.10 | 3.07 | n.d. | 0.096 | 17.50 | 0.00 | 2.89 | 12.36 |

**Table1: Average data for anaerobic growth and by-product synthesis under aerobic conditions**

| **ANAEROBIC** | **MG1655**  **_(UQ.DMK.MK)_** | **AV34**  **_(UQ)_** | **AV33**  **_(DMK.MK)_** | **AV36**  **_(DMK)_** |
| --- | --- | --- | --- | --- |
| **Growth [h^-1^]** | 0.38 ± 0.07 | 0.05 ± 0.01 | 0.35 ± 0.02 | 0.37 ± 0.03 |
| **Organic acids** | **Yield [mol/mol_Glc_]** | | | |
| Formate | 1.21 ± 0.07 | 0.86 ± 0.20 | 1.32 ± 0.10 | 1.16 ± 0.14 |
| Pyruvate | 0.014 ± 0.002 | 0.03 ± 0.02 | 0.016 ± 0.004 | 0.012 ± 0.005 |
| Lactate | 0.39 ± 0.03 | 0.93 ± 0.03 | 0.32 ± 0.04 | 0.45 ± 0.11 |
| DHO | 0.0005 ± 0.0001 | 0.029 ± 0.007 | 0.0006 ± 0.0001 | 0.0008 ± 0.0001 |
| Orotate | 0.006 ± 0.002 | 0 | 0.004 ± 0.001 | 0.005 ± 0.0008 |
| Acetate | 0.71 ± 0.09 | 0.58 ± 0.00 | 0.67 ± 0.09 | 0.62 ± 0.06 |
| Fumarate | 0.0022 ± 0.0012 | 0.008 ± 0.001 | 0.004 ± 0.001 | 0.002 ± 0.001 |
| Succinate | 0.14 ± 0.036 | 0 | 0.16 ± 0.04 | 0.15 ± 0.02 |
| Ethanol | 0.65 ± 0.11 | 0.61 ± 0.08 | 0.77 ± 0.04 | 0.46 ± 0.08 |

This table summarizes the data from the different growth curves displayed in sections 1-4. The table indicates specific growth rates during the exponential phase of growth given in doublings per hour. Product yields are given in mol product produced per mol of glucose consumed. Yields of 0 mean that the respective product could not be detected throughout the time course of the measurements.
