# Supplementary material for: All three quinone species play distinct roles in ensuring optimal growth under aerobic and fermentative conditions in E. coli K12
Source: PLoS One. 2018 Apr 3;13(4):e0194699. doi: 10.1371/journal.pone.0194699 (PMC5882134; doi:10.1371/journal.pone.0194699)
Supplement: S1 Dataset — This file contains the following: Suppl_data_aerobe.docx: Time course data for biomass and by-products from aerobic growth experiments. Suppl_data_anaerobe.docx: Time course data for biomass and by-products from anaerobic growth experiments. Suppl_data_CellRox: Individual data from oxidative stress measurements. Suppl_data_Quinone_aerobe.docx: Individual quinone concentrations from aerobic growth experiments. Suppl_data_Quinone_anaerobe.docx: Individual quinone concentrations from anaerobic growth experiments. (ZIP) [file pone.0194699.s002.zip › Suppl_Data/Suppl_data_CellROX.docx]

**Table1: Measured meanX values from flow cytometry experiments with CellROX Green**

|  | **MG1655** | | **AV34** | | **AV33** | | **AV36** | |
| --- | --- | --- | --- | --- | --- | --- | --- | --- |
|  |  | | | | | | | |
| **Menadione [µM]** | 0 | 100 | 0 | 100 | 0 | 100 | 0 | 100 |
| 28.11.2017 | 0.4 | 1.77 | 0.36 |  |  |  |  |  |
| 05.12.2017 | 0.26 | 1.77 | 0.25 | 2.8 | 0.96 | 3.33 |  | 2.06 |
| 07.12.2017 | 0.24 |  | 0.24 |  | 0.74 |  | 0.38 | 1.25 |
| 13.12.2017 | 0.47 | 1.57 | 0.29 | 1.19 | 0.86 | 1.71 | 0.68 | 0.96 |
| 19.12.2017 |  |  |  | 1.64 | 0.61 | 1.14 | 0.55 | 0.93 |
| **Average** | 0.34 | 1.70 | 0.30 | 1.88 | 0.79 | 2.06 | 0.54 | 1.30 |
| **StDev** | 0.11 | 0.12 | 0.06 | 0.83 | 0.15 | 1.14 | 0.15 | 0.53 |

The table shows five repeats of the experiments with CellROX Green. Experiments were performed as indicated in Materials and Methods. The data represent the meanX (average fluorescence) values resulting from analyzing the fluorescence of single cells in FL1 (527/30) using the software of CyFlow Space.
