# Supplementary material for: All three quinone species play distinct roles in ensuring optimal growth under aerobic and fermentative conditions in E. coli K12
Source: PLoS One. 2018 Apr 3;13(4):e0194699. doi: 10.1371/journal.pone.0194699 (PMC5882134; doi:10.1371/journal.pone.0194699)
Supplement: S1 Dataset — This file contains the following: Suppl_data_aerobe.docx: Time course data for biomass and by-products from aerobic growth experiments. Suppl_data_anaerobe.docx: Time course data for biomass and by-products from anaerobic growth experiments. Suppl_data_CellRox: Individual data from oxidative stress measurements. Suppl_data_Quinone_aerobe.docx: Individual quinone concentrations from aerobic growth experiments. Suppl_data_Quinone_anaerobe.docx: Individual quinone concentrations from anaerobic growth experiments. (ZIP) [file pone.0194699.s002.zip › Suppl_Data/Suppl_data_Quinone aerobe.docx]

Table 1 Quinone distribution of MG1655 under aerobic conditions.

|  | **UQH [nmol/g]** | **UQ [nmol/g]** | **UQGes [nmol/g]** | **DMK [nmol/g]** | **MK [nmol/g]** | **Q Ges**  **[nmol/g]** |
| --- | --- | --- | --- | --- | --- | --- |
| **121023_MG1655_1a** | 516.94 | 622.37 | 1139.31 | 3.25 | 2.73 | 1145.29 |
| **121023_MG1655_1b** | 527.08 | 675.45 | 1202.53 | 8.20 | 2.14 | 1212.87 |
| **121023_MG1655_2a** | 396.64 | 627.38 | 1024.02 | 0.81 | 8.92 | 1033.74 |
| **121023_MG1655_2b** | 597.27 | 608.15 | 1205.42 | 1.18 | 33.94 | 1240.55 |
| **121023-MG1655_3a** | 640.96 | 572.89 | 1213.85 | 4.31 | 15.32 | 1233.48 |
| **121023_MG1655_4a** | 678.81 | 600.08 | 1278.89 | 24.49 | 24.95 | 1328.33 |
| **121023-MG1655_4b** | 619.49 | 554.82 | 1174.31 | 0.72 | 23.10 | 1198.13 |
| **130523_MG1655_1a+b** | 415.19 | 420.52 | 835.71 | 2.05 | 13.37 | 851.13 |
| **130523_MG1655_2a** | 366.92 | 527.17 | 894.09 | 43.04 | 8.82 | 945.96 |
| **130523_MG1655_3a** | 377.77 | 785.98 | 1163.75 | 2.70 | 37.86 | 1204.32 |
| **130523_MG1655_3b** | 367.31 | 761.22 | 1128.54 | 9.27 | 38.78 | 1176.59 |
| **170831_MG1655_A** | 235.16 | 769.97 | 1005.13 | 0.00 | 57.61 | 1062.74 |
| **170831_MG1655_B** | 260.80 | 653.66 | 914.46 | 0.00 | 59.58 | 974.04 |
| **Average [nmol/g]** | 461.57 | 629.20 | 1090.77 | 7.69 | 25.16 | 1123.63 |
| **STDEV** | 145.17 | 103.51 | 141.09 | 12.51 | 19.29 | 138.72 |
| **RATIO [%]** | 41 | 56 |  | 0.71 | 2.27 |  |
| **STDEV (Ratio) [%]** | 10 | 9 |  | 1.2 | 1.95 |  |

The values indicated in the table refer to quinone concentrations per g dry cell weight. Sampling and measurements were carried out as described in Material and Methods. Quinone concentration were calculated from the respective peak areas in the chromatogram. All quinone concentrations of a sample are determined from the same chromotogram. During the exponential growth phase of a single growth experiment multiple samples were taken. Dashed lines indicate independent experiments. The ratio indicated the percentage distribution of the quinone species in a strain. The total amount of all quinone species detected in one sample was set to 100 %.

Table 2 Quinone distribution of AV34 under aerobic conditions.

|  | **UQH [nmol/g]** | **UQ [nmol/g]** | **UQGes [nmol/g]** | **DMK [nmol/g]** | **MK [nmol/g]** | **Q Ges [nmol/g]** |
| --- | --- | --- | --- | --- | --- | --- |
| **130523a_AV34_1a+b** | 500.36 | 556.54 | 1056.90 | 0.00 | 0.00 | 1056.90 |
| **130523a_AV34_2a** | 666.64 | 469.42 | 1136.06 | 0.00 | 0.00 | 1136.06 |
| **130523a_AV34_2b** | 578.98 | 496.14 | 1075.11 | 0.00 | 0.00 | 1075.11 |
| **130523a_AV34_3a** | 320.61 | 1001.76 | 1322.37 | 0.00 | 0.00 | 1322.37 |
| **130523a_AV34_3b** | 363.16 | 986.46 | 1349.63 | 0.00 | 0.00 | 1349.63 |
| **130523b_AV34_1a+b** | 481.21 | 541.45 | 1022.66 | 0.00 | 0.00 | 1022.66 |
| **130523b_AV34_2a** | 292.01 | 672.77 | 964.78 | 0.00 | 0.00 | 964.78 |
| **130523b_AV34_2b** | 292.62 | 715.30 | 1007.93 | 0.00 | 0.00 | 1007.93 |
| **130523b_AV34_3a** | 575.53 | 550.40 | 1125.93 | 0.00 | 0.00 | 1125.93 |
| **130523b_AV34_3b** | 665.16 | 607.08 | 1272.23 | 0.00 | 0.00 | 1272.23 |
| **130611_AV34_1b** | 560.16 | 738.57 | 1298.73 | 0.00 | 0.00 | 1298.73 |
| **130611_AV34_2a** | 289.87 | 690.49 | 980.36 | 0.00 | 0.00 | 980.36 |
| **130611_AV34_2b** | 277.41 | 677.66 | 955.07 | 0.00 | 0.00 | 955.07 |
| **130611_AV34_3a** | 257.55 | 461.92 | 719.47 | 0.00 | 0.00 | 719.47 |
| **130611_AV34_3b** | 320.92 | 457.45 | 778.36 | 0.00 | 0.00 | 778.36 |
| **130611_AV34_4a** | 688.55 | 405.63 | 1094.18 | 0.00 | 0.00 | 1094.18 |
| **130611_AV34_4b** | 390.53 | 239.13 | 629.66 | 0.00 | 0.00 | 629.66 |
| **Average [nmol/g]** | 442.43 | 604.01 | 1046.44 | 0.00 | 0.00 | 1046.44 |
| **STDEV [nmol/g]** | 154.89 | 194.55 | 205.27 | 0.00 | 0.00 | 205.27 |
| **RATIO [%]** | 43 | 55 |  | 0 | 0 |  |
| **STDEV (Ratio) [%]** | 13 | 13 |  | 0 | 0 |  |

The values indicated in the table refer to quinone concentrations per g dry cell weight. Sampling and measurements were carried out as described in Material and Methods. Quinone concentration were calculated from the respective peak areas in the chromatogram. All quinone concentrations of a sample are determined from the same chromotogram. During the expoential growth phase of a single growth experiment multiple samples were taken. Dashed lines indicate independent experiments. The ratio indicates the percentage distribution of the quinone species in a strain. The total amount of all squinone species detected in one sample was set to 100 %.

Table 3 Quinone distribution of AV33 under aerobic conditions.

|  | **UQH [nmol/g]** | **UQ [nmol/g]** | **UQGes [nmol/g]** | **DMK [nmol/g]** | **MK [nmol/g]** | **Q Ges [nmol/g]** |
| --- | --- | --- | --- | --- | --- | --- |
| **121018_AV33_1a+b** | 0.00 | 0.00 | 0.00 | 415.83 | 556.76 | 972.59 |
| **121018_AV33_1a+b** | 0.00 | 0.00 | 0.00 | 452.34 | 636.55 | 1088.89 |
| **121018_AV33_2a+b** | 0.00 | 0.00 | 0.00 | 355.32 | 649.00 | 1004.32 |
| **121018_AV33_3a+b** | 0.00 | 0.00 | 0.00 | 361.65 | 539.54 | 901.20 |
| **121018_AV33_4a+b** | 0.00 | 0.00 | 0.00 | 413.15 | 553.76 | 966.91 |
| **130806_AV33_1A** | 0.00 | 0.00 | 0.00 | 533.14 | 611.00 | 1144.14 |
| **130806_AV33_1B** | 0.00 | 0.00 | 0.00 | 598.97 | 574.97 | 1173.93 |
| **130806_AV33_2A** | 0.00 | 0.00 | 0.00 | 491.46 | 515.22 | 1006.68 |
| **130806_AV33_2B** | 0.00 | 0.00 | 0.00 | 564.51 | 479.58 | 1044.10 |
| **130806_AV33_3A** | 0.00 | 0.00 | 0.00 | 481.08 | 426.33 | 907.42 |
| **130806_AV33_3B** | 0.00 | 0.00 | 0.00 | 484.75 | 409.37 | 894.12 |
| **130806_AV33_A** | 0.00 | 0.00 | 0.00 | 373.58 | 283.38 | 656.96 |
| **130806_AV33_B** | 0.00 | 0.00 | 0.00 | 374.47 | 273.76 | 648.23 |
| **130912_AV33_A** | 0.00 | 0.00 | 0.00 | 283.97 | 243.40 | 527.38 |
| **130912_AV33_B** | 0.00 | 0.00 | 0.00 | 290.61 | 230.09 | 520.70 |
| **Average [nmol/g]** | 0.00 | 0.00 | 0.00 | 431.66 | 465.52 | 897.17 |
| **STDEV [nmol/g]** | 0.00 | 0.00 | 0.00 | 94.45 | 146.31 | 211.84 |
| **RATIO [%]** | 0 | 0 |  | 49 | 51 |  |
| **STDEV (Ratio) [%]** | 0 | 0 |  | 7 | 7 |  |

The values indicated in the table refer to quinone concentrations per g dry cell weight. Sampling and measurements were carried out as described in Material and Methods. Quinone concentration were calculated from the respective peak areas in the chromatogram. All quinone concentrations of a sample are determined from the same chromotogram. During the expoential growth phase of a single growth experiment multiple samples were taken. Dashed lines indicate independent experiments. The ratio indicates the percentage distribution of the quinone species in a strain. The total amount of all squinone species detected in one sample was set to 100 %.

Table 4 Quinone distribution of AV36 under aerobic conditions.

|  | **UQH [nmol/g]** | **UQ [nmol/g]** | **UQGes [nmol/g]** | **DMK [nmol/g]** | **MK [nmol/g]** | **Q Ges [nmol/g]** |
| --- | --- | --- | --- | --- | --- | --- |
| **130806_AV36_A** | 0.00 | 0.00 | 0.00 | 337.70 | 0.00 | 337.70 |
| **130806_AV36_B** | 0.00 | 0.00 | 0.00 | 306.72 | 0.00 | 306.72 |
| **131014_AV36_2A** | 0.00 | 0.00 | 0.00 | 464.06 | 0.00 | 464.06 |
| **131014_AV36_2B** | 0.00 | 0.00 | 0.00 | 493.12 | 0.00 | 493.12 |
| **131014_AV36_3A** | 0.00 | 0.00 | 0.00 | 469.40 | 0.00 | 469.40 |
| **131014_AV36_3B** | 0.00 | 0.00 | 0.00 | 426.89 | 0.00 | 426.89 |
| **130912_AV36_A** | 0.00 | 0.00 | 0.00 | 486.92 | 0.00 | 486.92 |
| **130912_AV36_B** | 0.00 | 0.00 | 0.00 | 342.44 | 0.00 | 342.44 |
| **Average** | **0.00** | **0.00** | **0.00** | **415.90** | **0.00** | **415.90** |
| **STDEV** | **0.00** | **0.00** | **0.00** | **75.35** | **0.00** | **75.35** |
| **RATIO [%]** | **0** | **0** |  | **100** | **0** |  |
| **STDEV(Ratio) [%]** | **0** | **0** |  | **0** | **0** |  |

The values indicated in the table refer to quinone concentrations per g dry cell weight. Sampling and measurements were carried out as described in Material and Methods. Quinone concentration were calculated from the respective peak areas in the chromatogram. All quinone concentrations of a sample are determined from the same chromotogram. During the expoential growth phase of a single growth experiment multiple samples were taken. Dashed lines indicate independent experiments. The ratio indicates the percentage distribution of the quinone species in a strain. The total amount of all squinone species detected in one sample was set to 100 %.

Figure 1 Quinone content of the wildtype MG1655 and the mutants during exponential growth under aerobic batch conditions. The figure summarizes the data of Tab- 1-4 and shows the average quinone content of the different strains.

Table 5 Relative quinone distribution of the wildtype MG1655 and the mutants under aerobic batch conditions.

| **Ratio [%)** | **MG1655** | **AV34** | **AV33** | **AV36** |
| --- | --- | --- | --- | --- |
| **UQH** | 41 ± 10 | 43 ± 13 | 0 ± 0 | 0 ± 0 |
| **UQ** | 56 ± 9 | 57 ± 13 | 0 ± 0 | 0 ± 0 |
| **DMK** | 0.71 ± 1.2 | 0 ± 0 | 49 ± 7 | 100 ± 0 |
| **MK** | 2.24 ± 1.9 | 0 ± 0 | 51 ± 7 | 0 ± 0 |

The table summarizes the precentage distribution of the quinone species obtained from Tab- 1-4.
