# Supplementary material for: All three quinone species play distinct roles in ensuring optimal growth under aerobic and fermentative conditions in E. coli K12
Source: PLoS One. 2018 Apr 3;13(4):e0194699. doi: 10.1371/journal.pone.0194699 (PMC5882134; doi:10.1371/journal.pone.0194699)
Supplement: S1 Dataset — This file contains the following: Suppl_data_aerobe.docx: Time course data for biomass and by-products from aerobic growth experiments. Suppl_data_anaerobe.docx: Time course data for biomass and by-products from anaerobic growth experiments. Suppl_data_CellRox: Individual data from oxidative stress measurements. Suppl_data_Quinone_aerobe.docx: Individual quinone concentrations from aerobic growth experiments. Suppl_data_Quinone_anaerobe.docx: Individual quinone concentrations from anaerobic growth experiments. (ZIP) [file pone.0194699.s002.zip › Suppl_Data/Suppl_data_Quinone anaerobe.docx]

Table 1 Quinone distribution of MG1655 under anaerobic conditions.

|  | **UQH** | **UQ** | **DMK** | **MK** | Ges Q |
| --- | --- | --- | --- | --- | --- |
|  | **nmol/g** | **nmol/g** | **nmol/g** | **nmol/g** | nmol/g |
| **161011_MG1655_1A** | 0.00 | 175.21 | 194.80 | 450.74 | 820.75 |
| **161011_MG1655_1B** | 0.00 | 181.97 | 151.62 | 385.78 | 719.37 |
| **161011_MG1655_2A** | 0.00 | 174.71 | 94.21 | 300.62 | 569.54 |
| **161011_MG1655_2B** | 0.00 | 141.44 | 102.38 | 247.41 | 491.22 |
| **161011_MG1655_3A** | 0.00 | 126.09 | 366.56 | 237.17 | 729.82 |
| **161011_MG1655_3B** | 0.00 | 145.69 | 383.48 | 284.32 | 813.49 |
| **170109_MG1655_1A** | 0.00 | 172.21 | 162.21 | 276.23 | 610.64 |
| **170109_MG1655_1B** | 0.00 | 103.25 | 156.51 | 314.51 | 574.26 |
| **170109_MG1655_2A** | 0.00 | 147.13 | 133.01 | 311.09 | 591.23 |
| **170109_MG1655_2B** | 0.00 | 158.51 | 142.67 | 302.87 | 604.06 |
| **170109_MG1655_3A** | 0.00 | 131.82 | 77.66 | 236.47 | 445.95 |
| **170109_MG1655_3B** | 0.00 | 113.53 | 105.26 | 265.70 | 484.49 |
| **170822_MG1655_A** | 0.00 | 298.39 | 142.83 | 314.62 | 755.83 |
| **170822_MG1655_B** | 0.00 | 348.44 | 172.00 | 357.90 | 878.34 |
| **Average [nmol/g]** | **0.00** | **161.23** | **159.01** | **285.69** | **605.93** |
| **STDEV** | **0.00** | **79.89** | **99.42** | **97.71** | **213.36** |
| **Ratio [%]** | **0** | **25** | **24** | **45** |  |
| **STDEV (Ratio) [%]** | **0** | **10** | **12** | **14** |  |

The values indicated in the table refer to quinone concentrations per g dry cell weight. Sampling and measurements were carried out as described in Material and Methods. Quinone concentration were calculated from the respective peak areas in the chromatogram. All quinone concentrations of a sample are determined from the same chromotogram. During the exponential growth phase of a single growth experiment multiple samples were taken. Dashed lines indicate independent experiments.

Table 2 Quinone distribution of AV34 under anaerobic conditions.

|  | **UQH** | **UQ** | **DMK** | **MK** | **Ges Q** |
| --- | --- | --- | --- | --- | --- |
|  | **nmol/g** | **nmol/g** | **nmol/g** | **nmol/g** | **nmol/g** |
| **161123_AV34_1A** | 0.00 | 828.03 | 0.00 | 0.00 | 828.03 |
| **161123_AV34_1B** | 0.00 | 929.16 | 0.00 | 0.00 | 929.16 |
| **161123_AV34_2A** | 0.00 | 315.73 | 0.00 | 0.00 | 315.73 |
| **170117_AV34_1A** | 0.00 | 611.69 | 0.00 | 0.00 | 611.69 |
| **170117_AV34_1B** | 0.00 | 690.80 | 0.00 | 0.00 | 690.80 |
| **170117_AV34_2A** | 0.00 | 685.60 | 0.00 | 0.00 | 685.60 |
| **170117_AV34_2B** | 0.00 | 698.58 | 0.00 | 0.00 | 698.58 |
| **170706_AV34_1A** | 0.00 | 560.38 | 0.00 | 0.00 | 560.38 |
| **170706_AV34_2A** | 0.00 | 887.46 | 0.00 | 0.00 | 887.46 |
| **170706_AV34_1B** | 0.00 | 523.66 | 0.00 | 0.00 | 523.66 |
| **170706_AV34_2B** | 0.00 | 613.10 | 0.00 | 0.00 | 613.10 |
| **170829_AV34_A** | 0.00 | 437.49 | 0.00 | 0.00 | 437.49 |
| **170829_AV34_B** | 0.00 | 357.06 | 0.00 | 0.00 | 357.06 |
| **Average [nmol/g]** | **0.00** | **581.34** | **0.00** | **0.00** | **581.34** |
| **STDEV** | **0.00** | **190.04** | **0.00** | **0.00** | **190.04** |
| **Ratio [%]** | **0** | **100** | **0** | **0** |  |
| **STDEV (Ratio) [%]** | **0** | **0** | **0** | **0** |  |

The values indicated in the table refer to quinone concentrations per g dry cell weight. Sampling and measurements were carried out as described in Material and Methods. Quinone concentration were calculated from the respective peak areas in the chromatogram. All quinone concentrations of a sample are determined from the same chromotogram. During the exponential growth phase of a single growth experiment multiple samples were taken. Dashed lines indicate independent experiments.

Table 3 Quinone distribution of AV33 under anaerobic conditions.

|  | **UQH** | **UQ** | **DMK** | **MK** | **Ges Q** |
| --- | --- | --- | --- | --- | --- |
|  | **nmol/g** | **nmol/g** | **nmol/g** | **nmol/g** | **nmol/g** |
| **161103_AV33_1A** | 0.00 | 0.00 | 208.70 | 376.23 | 584.93 |
| **161103_AV33_1B** | 0.00 | 0.00 | 144.37 | 346.55 | 490.91 |
| **161103_AV33_2A** | 0.00 | 0.00 | 76.83 | 166.96 | 243.79 |
| **170216_AV33_1A** | 0.00 | 0.00 | 194.37 | 444.77 | 639.14 |
| **170216_AV33_1B** | 0.00 | 0.00 | 230.00 | 427.49 | 657.49 |
| **170216_AV33_2A** | 0.00 | 0.00 | 213.11 | 514.10 | 727.22 |
| **170216_AV33_1A** | 0.00 | 0.00 | 273.16 | 549.15 | 822.31 |
| **170216_AV33_1B** | 0.00 | 0.00 | 341.55 | 589.48 | 931.03 |
| **170216_AV33_2A** | 0.00 | 0.00 | 350.92 | 690.49 | 1041.41 |
| **141029_AV33_1A** | 0.00 | 0.00 | 296.36 | 362.25 | 658.60 |
| **141029_AV33_1B** | 0.00 | 0.00 | 330.79 | 334.77 | 665.56 |
| **141029_AV33_2A** | 0.00 | 0.00 | 296.47 | 377.52 | 673.99 |
| **141029_AV33_2B** | 0.00 | 0.00 | 312.31 | 407.52 | 719.83 |
| **141029_AV33_3A** | 0.00 | 0.00 | 251.37 | 402.18 | 653.55 |
| **141029_AV33_3B** | 0.00 | 0.00 | 241.81 | 403.23 | 645.04 |
| **150218_AV33_1A** | 0.00 | 0.00 | 230.65 | 295.28 | 525.93 |
| **150218_AV33_1B** | 0.00 | 0.00 | 293.45 | 277.40 | 570.85 |
| **150218_AV33_2A** | 0.00 | 0.00 | 177.82 | 166.08 | 343.91 |
| **150218_AV33_2B** | 0.00 | 0.00 | 163.01 | 196.97 | 359.98 |
| **150218_AV33_3A** | 0.00 | 0.00 | 258.69 | 333.61 | 592.30 |
| **150218_AV33_3B** | 0.00 | 0.00 | 249.03 | 287.33 | 536.37 |
| **Average [nmol/g]** | **0.00** | **0.00** | **244.51** | **378.54** | **623.05** |
| **STDEV** | **0.00** | **0.00** | **69.31** | **132.60** | **182.87** |
| **Ratio** | **0** | **0** | **40** | **60** |  |
| **STDEV (Ratio) [%]** | **0** | **0** | **7** | **7** |  |

The values indicated in the table refer to quinone concentrations per g dry cell weight. Sampling and measurements were carried out as described in Material and Methods. Quinone concentration were calculated from the respective peak areas in the chromatogram. All quinone concentrations of a sample are determined from the same chromotogram. During the exponential growth phase of a single growth experiment multiple samples were taken. Dashed lines indicate independent experiments.

Table 4 Quinone distribution of MG1655 under anaerobic conditions.

|  | **UQH** | **UQ** | **DMK** | **MK** | **Ges Q** |
| --- | --- | --- | --- | --- | --- |
|  | **nmol/g** | **nmol/g** | **nmol/g** | **nmol/g** | **nmol/g** |
| **161011_AV36_1A** | 0.00 | 0.00 | 427.17 | 0.00 | 427.17 |
| **161011_AV36_1B** | 0.00 | 0.00 | 365.22 | 0.00 | 365.22 |
| **161011_AV36_2A** | 0.00 | 0.00 | 533.24 | 0.00 | 533.24 |
| **161011_AV36_2B** | 0.00 | 0.00 | 524.83 | 0.00 | 524.83 |
| **161011_AV36_3A** | 0.00 | 0.00 | 515.74 | 0.00 | 515.74 |
| **161011_AV36_3B** | 0.00 | 0.00 | 608.46 | 0.00 | 608.46 |
| **170124_AV36_1A** | 0.00 | 0.00 | 291.47 | 0.00 | 291.47 |
| **170124_AV36_1B** | 0.00 | 0.00 | 292.86 | 0.00 | 292.86 |
| **170124_AV36_2A** | 0.00 | 0.00 | 465.31 | 0.00 | 465.31 |
| **170124_AV36_2B** | 0.00 | 0.00 | 469.53 | 0.00 | 469.53 |
| **170124_AV36_3A** | 0.00 | 0.00 | 190.77 | 0.00 | 190.77 |
| **170124_AV36_3B** | 0.00 | 0.00 | 238.30 | 0.00 | 238.30 |
| **141203_AV36_1A** | 0.00 | 0.00 | 328.06 | 0.00 | 328.06 |
| **141203_AV36_1B** | 0.00 | 0.00 | 307.03 | 0.00 | 307.03 |
| **141203_AV36_2A** | 0.00 | 0.00 | 442.55 | 0.00 | 442.55 |
| **141203_AV36_2B** | 0.00 | 0.00 | 448.50 | 0.00 | 448.50 |
| **141203_AV36_3A** | 0.00 | 0.00 | 350.34 | 0.00 | 350.34 |
| **141203_AV36_3B** | 0.00 | 0.00 | 383.40 | 0.00 | 383.40 |
| **150210_AV36_1A** | 0.00 | 0.00 | 333.55 | 0.00 | 333.55 |
| **150210_AV36_1B** | 0.00 | 0.00 | 398.67 | 0.00 | 398.67 |
| **150210_AV36_2A** | 0.00 | 0.00 | 576.37 | 0.00 | 576.37 |
| **150210_AV36_2B** | 0.00 | 0.00 | 503.89 | 0.00 | 503.89 |
| **150210_AV36_3A** | 0.00 | 0.00 | 624.78 | 0.00 | 624.78 |
| **150210_AV36_3B** | 0.00 | 0.00 | 502.90 | 0.00 | 502.90 |
| **Average [nmol/g]** | **0.00** | **0.00** | **421.79** | **0.00** | **421.79** |
| **STDEV** | **0.00** | **0.00** | **116.32** | **0.00** | **116.32** |
| **Ratio [%]** | **0** | **0** | **100** | **0** |  |
| **STDEV (Ratio) [%]** | **0** | **0** | **0** | **0** |  |

The values indicated in the table refer to quinone concentrations per g dry cell weight. Sampling and measurements were carried out as described in Material and Methods. Quinone concentration were calculated from the respective peak areas in the chromatogram. All quinone concentrations of a sample are determined from the same chromotogram. During the exponential growth phase of a single growth experiment multiple samples were taken. Dashed lines indicate independent experiments.

Figure 1 Quinone content of the wildtype MG1655 and the mutants during exponential growth under anaerobic batch conditions without an alternative electron acceptor. The figure shows the average data obtained from the measurement data referenced in Tab. 1-4.

**Table 5 Relative quinone distribution [%] of the wildtype MG1655 and the mutants under anaerobic batch conditions without an alternative electron acceptor.**

| **Ratio [%)** | **MG1655** | **AV34** | **AV33** | **AV36** |
| --- | --- | --- | --- | --- |
| **UQH** | 0 ± 0 | 0 ± 0 | 0 ± 0 | 0 ± 0 |
| **UQ** | 25 ± 10 | 100 ± 0 | 0 ± 0 | 0 ± 0 |
| **DMK** | 24 ±12 | 0 ± 0 | 40 ±7 | 100 ± 0 |
| **MK** | 45 ± 14 | 0 ± 0 | 60 ± 7 | 0 ± 0 |

The table shows the average values of the data points from Tab.1-4. The total amount of all quinone species detected in one sample was set to 100%.
